# The prevalence of antimicrobial resistance of bacterial strains from large-scale healthy chicken flocks in the Észak-Alföld region of Hungary

**DOI:** 10.3389/fvets.2025.1709725

**Published:** 2026-01-12

**Authors:** Ádám Kerek, Ábel Szabó, Franciska Barnácz, Bence Csirmaz, Ákos Jerzsele

**Affiliations:** 1 Department of Pharmacology and Toxicology, University of Veterinary Medicine Budapest, Budapest, Hungary; 2 National Laboratory of Infectious Animal Diseases, Antimicrobial Resistance, Veterinary Public Health and Food Chain Safety, University of Veterinary Medicine Budapest, Budapest, Hungary

**Keywords:** antimicrobial, chickens, *Enterococcus*, *Escherichia coli*, Hungary, MDR, minimum inhibitory concentration

## Abstract

**Introduction:**

Antimicrobial resistance (AMR) represents one of the major public health challenges of the 21st century, with profound implications for both human and veterinary medicine. Isolates recovered from clinically healthy birds from food-producing animals play a critical role as reservoirs and potential vectors for resistance genes, underscoring the importance of continuous surveillance. This study aimed to evaluate the phenotypic resistance profiles of *Staphylococcus aureus*, *Enterococcus* spp. and *Escherichia coli* isolated from large-scale chicken flocks in the Észak-Alföld region of Hungary.

**Methods:**

A total of 24 flocks were sampled, and bacterial isolates were subjected to antimicrobial susceptibility testing using the broth microdilution method. Minimum inhibitory concentrations (MICs) were interpreted according to European Committee on Antimicrobial Susceptibility Testing (EUCAST) and Clinical Laboratory Standard Institute (CLSI) guidelines.

**Results:**

Extremely high (>70.0%) levels of resistance were detected against doxycycline, amoxicillin, and florfenicol, while resistance to critically important antimicrobials such as ceftriaxone, imipenem and vancomycin remained comparatively low (1.0–10.0%) or rare (<0.1%). Multidrug resistance (MDR), defined as resistance to three or more antimicrobial classes, was observed in more than half of the isolates. Resistance patterns varied across farms, but amoxicillin and florfenicol resistance frequently co-occurred, serving as strong predictors of MDR status.

**Discussion:**

These findings highlight the substantial prevalence of AMR among bacteria in intensive poultry systems in Hungary. The results emphasize the need for enhanced antimicrobial stewardship, prudent antibiotic use in livestock, and targeted biosecurity interventions to mitigate the potential spillover of resistance into the broader ecosystem and human health. While the species investigated are known to include pathogenic strains, the isolates analyzed in this study were obtained from healthy birds and are thus regarded as isolates recovered from clinically healthy birds’ representatives.

## 1 Introduction

Antimicrobial resistance (AMR) is recognized as one of the foremost public health challenges of the 21st century, arising when genetic changes in bacteria reduce the efficacy of antibiotics used to treat infections. A UK-government commissioned report projected that AMR could cause up to 10 million deaths annually by 2050 (1). Although some experts have debated these estimates (2) both the World Health Organization (WHO) and numerous research groups agree that the global spread of AMR is an urgent problem requiring coordinated international action (3, 4). Monitoring the current prevalence of AMR, regional trends, and the susceptibility of key bacterial strains is essential.

In this context, region-specific data are particularly important to guide local interventions and surveillance strategies. The Észak-Alföld region of Hungary, characterized by its dense poultry farming and limited recent AMR data, offers a critical but underrepresented case for such targeted investigation.

Without effective interventions, including the widespread adoption of alternative approaches such as probiotics (5), plant essential oils and extracts (6–9), antimicrobial peptides (10), and other novel strategies (11, 12), the continued spread of resistance is likely to have increasingly severe consequences in the future.

In poultry production, *Enterococcus faecium* and *Enterococcus faecalis* are the most prevalent *Enterococcus* species associated with disease (13). They are particularly important contributors to chick mortality (14), while in adult birds they can cause bacteremia, pulmonary hypertension, amyloid arthropathy, and neurological disorders (13). Primary transmission routes include fecally contaminated eggs as well as aerosol and oral exposure (15). While *E. faecalis* is widely distributed, *E. faecium* is less common but typically exhibits higher levels of antibiotic resistance (16). Other *Enterococcus* species, including *E. hirae*, *E. durans*, and *E. casseliflavus*, have also been identified in poultry flocks (17–19), where they can induce diseases such as enteritis and septicemia, raising both veterinary and public health concerns due to their role in resistance dissemination (20, 21).

*Staphylococcus aureus* is another major pathogen in poultry, contributing to foodborne illness and often associated with fecal contamination of poultry-derived products (22). It poses zoonotic risks through transmission along the food chain. Selective pressure from antibiotic use in bacteria such as *Escherichia coli* and *S. aureus* facilitates the emergence and dissemination of AMR (23, 24). Through horizontal gene transfer, these bacteria can spread resistance genes and plasmids across animal and human populations, exacerbating the threat of drug resistance (25).

Certain *S. aureus* strains produce enterotoxins which can cause food poisoning in humans, as well as a variety of extracellular toxins (26). Because enterotoxins are heat-stable, they can persist in food even after thermal processing (27), making *S. aureus* one of the most common sources of foodborne disease worldwide (28).

*Escherichia coli* represents another critical bacterial group, functioning both as a component of the gut microbiota and as a potential pathogen (29). In poultry, *E. coli* strains can cause colibacillosis, meningitis, diarrhea, and septicemia, resulting in considerable economic losses (30). Based on pathogenic classification, *E. coli* strains can be grouped as enterohemorrhagic, enterotoxigenic, enteropathogenic, enteroaggregative, or enteroinvasive (31). In addition, they are divided into isolates recovered from clinically healthy birds, intraintestinal pathogenic, and extraintestinal pathogenic types depending on their virulence profiles, which include toxins, hemolysins, siderophores, and adhesion factors (32). These determinants are central to disease progression.

Because *E. coli* reflects antibiotic usage patterns and the development of resistance within the gut microbiome, it is a cornerstone organism for AMR surveillance programs (33). Continuous monitoring of these bacteria is therefore critical, not only at national but also at regional levels. The present study aims to characterize the resistance profiles of bacteria isolated from large-scale layer flocks in the Észak-Alföld (North Great Plain) region of Hungary. By providing detailed regional data, this work seeks to contribute to a deeper understanding of AMR dynamics and to inform food safety strategies.

## 2 Materials and methods

### 2.1 Origin of samples

During a nationwide survey conducted between 2022 and 2023, isolates recovered from clinically healthy birds of *S. aureus*, *Enterococcus*, and *E. coli* were collected from seven regions of Hungary, including a total of 24 large-scale poultry farms (with at least three farms sampled per region). In accordance with Hungarian regulations, a large-scale poultry farm is defined as a facility housing at least 10,000 laying hens or 40,000 birds in total under intensive production. All bacterial isolates were collected from clinically healthy chickens without any signs of disease at the time of sampling and are therefore considered isolates recovered from clinically healthy birds in origin. The present manuscript focuses exclusively on data derived from the Észak-Alföld region, based on isolates obtained from three representative farms in that area. This regional subset was selected due to its significant poultry production and the limited availability of antimicrobial resistance (AMR) data from this part of the country. Sample collection was carried out with the consent of the farm owners and in collaboration with the Department of Animal Hygiene and Herd Health. Sampling was performed as part of a proactive, non-diagnostic health surveillance effort, aimed at assessing the antimicrobial resistance status of poultry flocks under normal production conditions. At the time of sampling, birds showed no clinical signs of disease and were considered clinically healthy based on the attending veterinarians’ routine health checks. All sampling procedures were conducted in accordance with national veterinary guidelines, and all farms participated anonymously.

From each farm, 15 tracheal and 15 cloacal swabs were collected using Amies-type transport swabs without charcoal and with standard aluminum shafts (Biolab Zrt., Budapest, Hungary). Species isolation was performed at the Microbiology Laboratory of the Department of Pharmacology and Toxicology, University of Veterinary Medicine Budapest. *S. aureus* isolates were cultured on CHROMagar™ Staph aureus (Chebio Fejlesztő Kft., Budapest, Hungary), *E. coli* isolates on ChromoBio® Coliform (Biolab Zrt., Budapest, Hungary), and *Enterococcus* isolates on m-*Enterococcus* Modified Agar (Merck KGaA, Darmstadt, Germany). For each target species, a single representative colony was selected per sample based on colony morphology and growth characteristics. This approach was used to avoid overrepresentation of clonal isolates.

Colonies with characteristic morphology and pigmentation were first selected for further analysis. Species identification was performed using MALDI-TOF mass spectrometry (Flextra-LAB Kft., Budapest, Hungary) with Biotyper software version 12.0 (Bruker Daltonics GmbH, Bremen, Germany, 2024) (34). Pure colonies were sub-cultured on tryptic soy agar (Biolab Zrt., Budapest, Hungary) and preserved as single cultures using the Microbank™ system (Pro-Lab Diagnostics, Richmond Hill, Canada) at −80 °C until further use. Each sample was assigned a unique identifier, and metadata including source tissue (trachea or cloaca), production type (breeding, layer, or broiler), bird age (grower or adult), and flock size (5,001–50,000; 50,001–100,000; >100,000) were recorded.

### 2.2 Determination of minimum inhibitory concentration (MIC)

Stock solutions of the tested antimicrobial agents (Merck KGaA, Darmstadt, Germany) were prepared according to Clinical Laboratory Standards Institute (CLSI) guidelines (35). Amoxicillin and amoxicillin-clavulanic acid (2:1 ratio) were dissolved in phosphate-buffered saline (pH 7.2, 0.01 mol/L), while imipenem was dissolved in phosphate buffer at pH 6.0 (0.1 mol/L). Ceftriaxone, doxycycline, spectinomycin, neomycin, colistin, tiamulin, tylosin, lincomycin, and vancomycin were dissolved in distilled water. For the sulphonamide-trimethoprim combination (19:1), sulfamethoxazole was dissolved in hot water with a few drops of 2.5 mol/L NaOH, while trimethoprim was dissolved in distilled water with 0.05 mol/L HCl. Enrofloxacin solutions were prepared in distilled water with a few drops of 1 mol/L NaOH, and florfenicol was dissolved in a mixture of 95% ethanol and distilled water.

For all agents, 1,024 μg/mL stock solutions were prepared, corrected for the manufacturer-specified purity of each compound. Antimicrobial susceptibility testing was performed by determining minimum inhibitory concentrations (MICs) using the broth microdilution method according to CLSI guidelines (VET06, 1st Edition). Epidemiological cutoff values and clinical breakpoints were defined in accordance with CLSI (35) and the European Committee on Antimicrobial Susceptibility Testing (EUCAST) guidelines v13.1 (36).

Bacterial strains stored in Microbank™ were subcultured in 3 mL Mueller-Hinton broth (MHB) and incubated at 37 °C for 18–24 h prior to testing. MIC determinations were performed in 96-well microtiter plates (VWR International Kft., Debrecen, Hungary). Except for the first column, each well was filled with 90 μL MHB. Stock solutions were serially diluted twofold from 512 μg/mL across columns 1–10. Wells in column 11 served as growth controls (inoculum only), while column 12 served as sterility controls (broth only). Inoculate were prepared to 0.5 McFarland standard, and 10 μL were dispensed into each test well. Plates were incubated at 37 °C for 18–24 h, and results were read using an SWIN automated MIC reader and the VIZION system (CheBio Fejlesztő Kft., Budapest, Hungary). Resistance rates were calculated as the percentage of isolates classified as resistant (based on established breakpoints) out of the total number of isolates tested for each antimicrobial agent. Species-specific percentages refer to the number of resistant isolates per bacterial species per compound. Human resistance data were provided by the Hungarian National Centre for Public Health and Pharmacy.

### 2.3 Statistical analysis

Statistical analyses were performed using R software version 4.1.0. Results from the Észak-Alföld region were binarized according to established antimicrobial susceptibility breakpoints to indicate resistance (1 = resistant, 0 = susceptible). Only antimicrobials with nonzero variance were retained for downstream analysis. Pairwise correlations between antimicrobials with nonzero variance were assessed using Spearman’s rank correlation with the Hmisc (v4.5–0) and corrplot (v0.92) packages. Principal component analysis (PCA) was applied to the binary resistance profiles to reduce dimensionality and identify dominant axes of variation among bacterial isolates. PCA and clustering were performed using the FactoMineR (v2.4) and factoextra (v1.0.7) packages, with cluster assignment based on the K-means algorithm (cluster v2.1.2), with the optimal number of clusters selected via the elbow method.

Multidrug resistance (MDR) was defined as resistance to three or more antimicrobial classes, and this binary MDR status was used as the outcome variable in decision tree models constructed in Python 3.8.10 using the DecisionTreeClassifier from scikit-learn (v0.24.2). Weighted network graphs were visualized with networkx (v2.5), while Monte Carlo simulations (*n* = 1,000 iterations) were performed using custom Python scripts and the numpy library (v1.20.3). The purpose was to test whether the observed MDR prevalence differed from what would be expected by chance.

The null hypothesis assumed that resistance to each antimicrobial was randomly distributed across isolates, while preserving the marginal resistance frequencies. This allowed estimation of the probability that the observed MDR frequency could occur under a random distribution model.

## 3 Results

**Figure 1:**
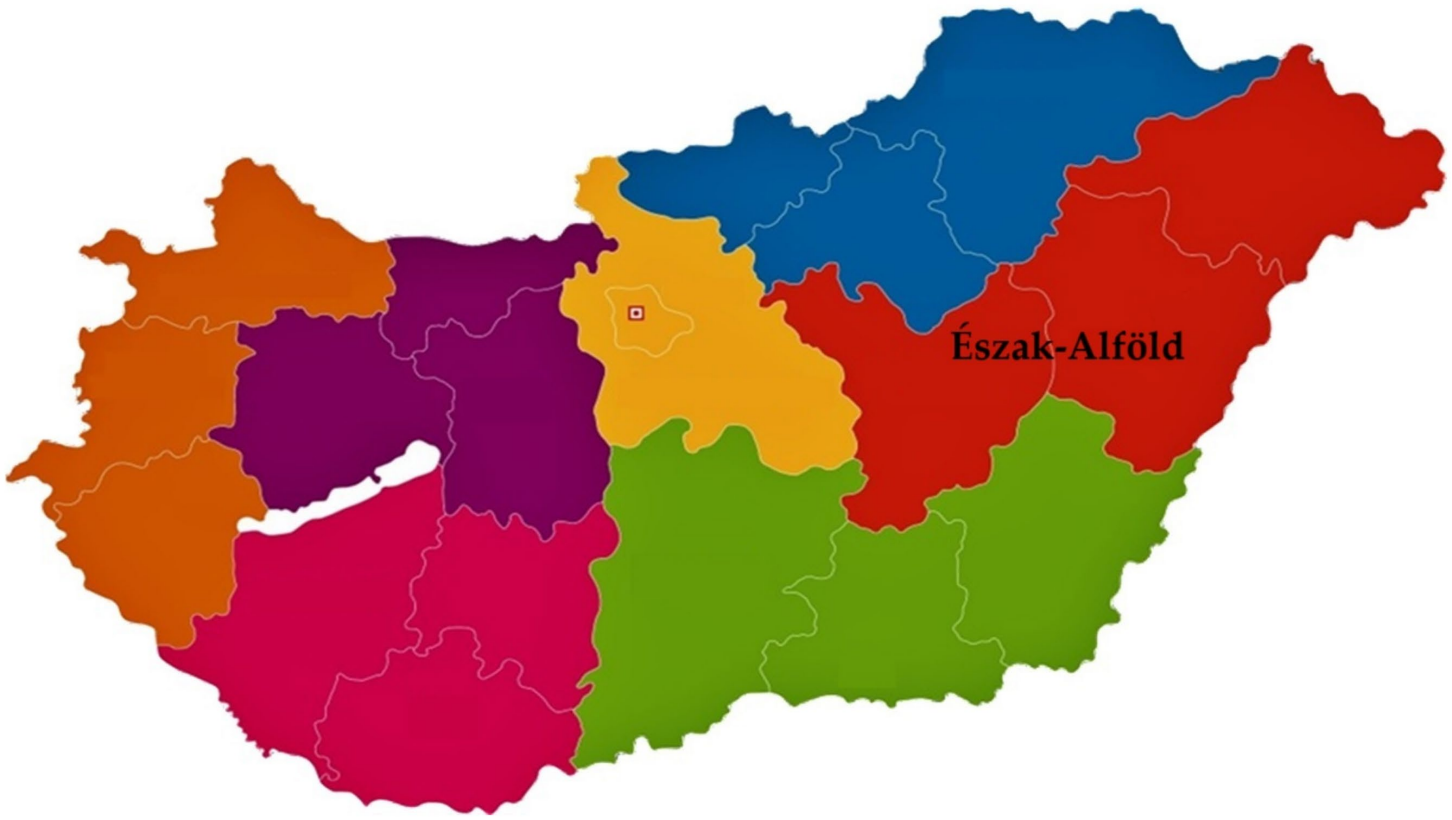
Map of Hungary showing its administrative regions, with the Észak-Alföld (North Great Plain) region highlighted in red. This study focused on large-scale chicken farms located within this region.

From the Észak-Alföld (North Great Plain) region (Figure 1), the antibiotic resistance profiles of *S. aureus* isolates (*n* = 22, representing 48.9% of the 45 samples) were evaluated against nine antimicrobial agents. Following classification into resistant or susceptible categories, multivariate statistical analyses, machine learning models, and simulation approaches were applied. Overall, 54.5% of isolates were identified as MDR.

In addition, *Enterococcus* isolates (*n* = 73, representing 81.1% of the 90 samples) from poultry samples were tested against 10 antibiotics using MIC determinations. Based on clinical breakpoints, 47 isolates (64.4%) were classified as MDR, being resistant to at least three antimicrobial agents.

Finally, *E. coli* isolates (*n* = 71, representing 78.9% of the 90 samples) were assessed for susceptibility to 11 antimicrobials. All isolates exhibited resistance to at least three agents, corresponding to an MDR prevalence of 100%.

### 3.1 *Staphylococcus aureus* isolates

**Figure 2:**
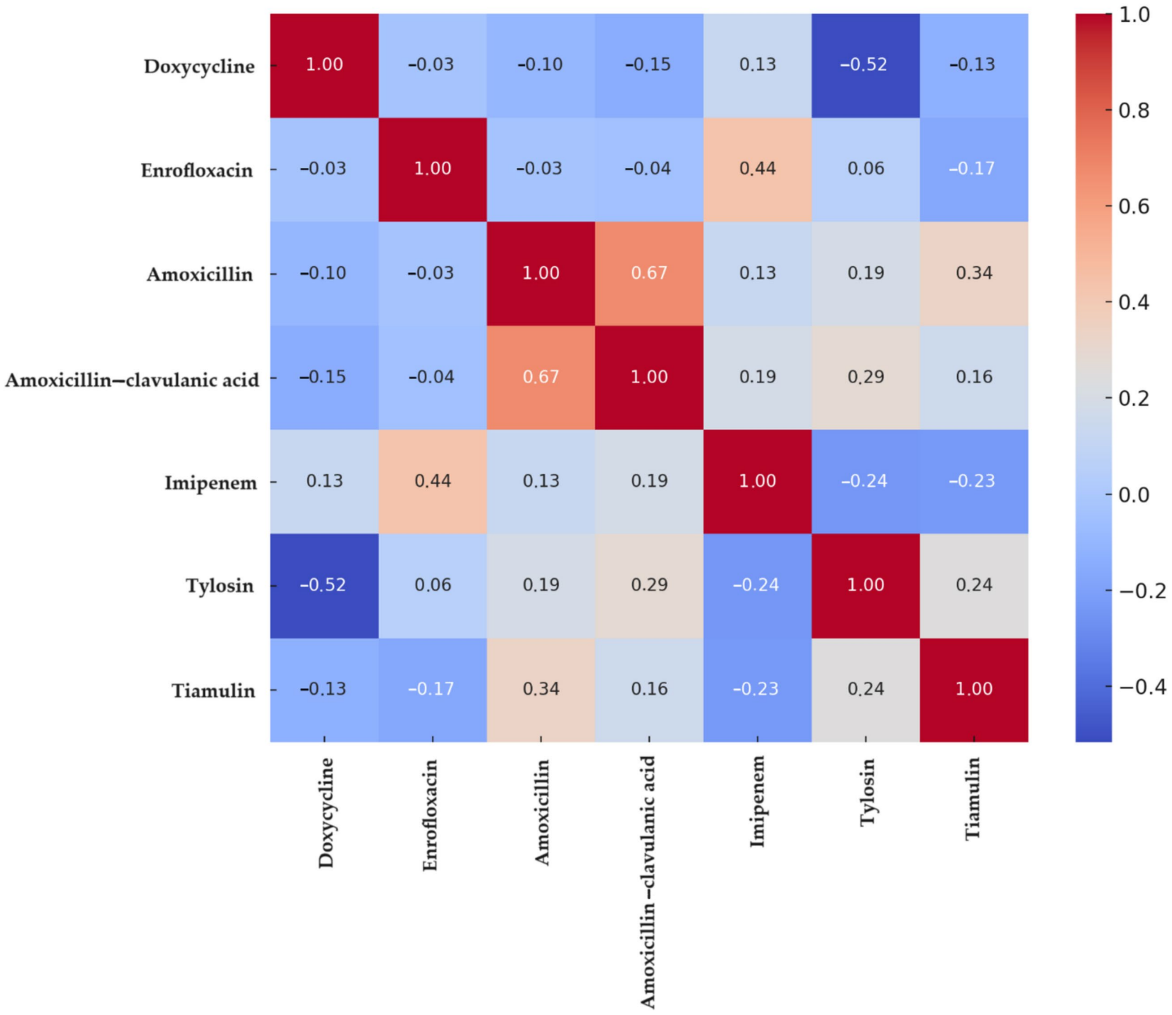
Spearman correlation heatmap of *Staphylococcus aureus* strains (*n* = 22) isolated from chickens in the Észak-Alföld region. A positive correlation indicates a higher probability of co-occurrence of resistance to both antimicrobial agents. A correlation value close to zero suggests that resistance to the two agents develops independently. A negative correlation implies that resistance to one agent may be associated with susceptibility to the other, indicating an inverse relationship.

Correlation analysis (Figure 2) excluded antimicrobials with zero variance—namely, potentiated sulphonamides (100% resistant) and vancomycin (100% susceptible). The strongest positive association was observed between amoxicillin and amoxicillin–clavulanic acid (0.67), followed by imipenem–enrofloxacin (0.44).

**Figure 3:**
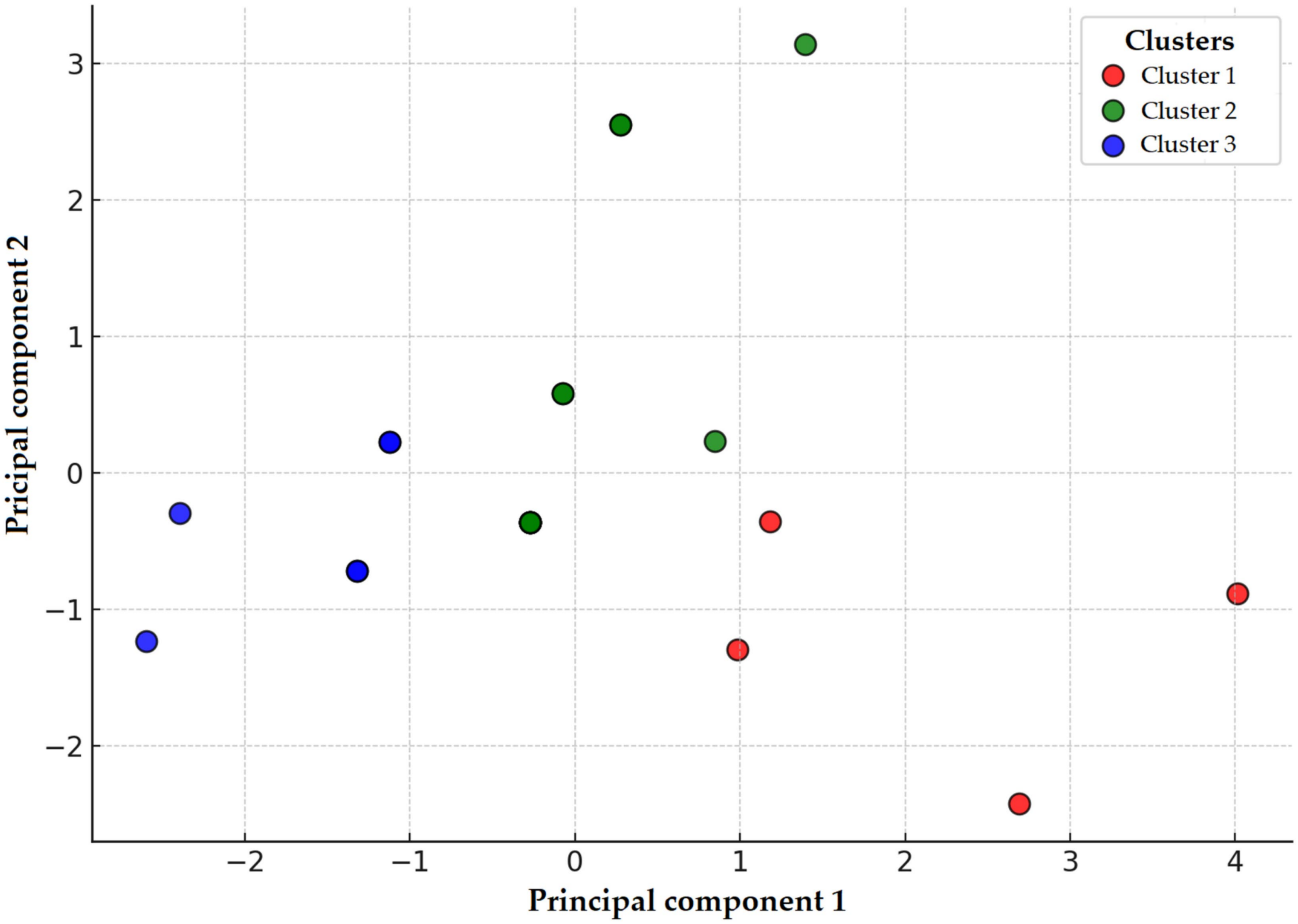
Principal component analysis (PCA) and clustering of *Staphylococcus aureus* strains (*n* = 22) isolated from chickens in the Észak-Alföld region. The red, green, and blue colors represent clusters 1, 2, and 3, respectively. Cluster 1 (red), characterized by extremely high-level resistance to doxycycline and potentiated sulphonamide; Cluster 2 (green), dominated by enrofloxacin resistance; and Cluster 3 (blue), showing elevated resistance to amoxicillin and amoxicillin–clavulanic acid. The spatial separation of clusters highlights underlying heterogeneity in resistance phenotypes, potentially linked to differences in antibiotic exposure, genetic backgrounds, or environmental factors. The clustering results also guide interpretation in subsequent decision tree and network graph analyses by illustrating distinct resistance pattern groupings.

PCA identified three distinct clusters reflecting divergent resistance profiles (Figure 3). These clusters were clearly separated along the principal components, indicating meaningful heterogeneity among isolates. Due to the limited sample size, a decision tree model could not be reliably developed for this species.

**Figure 4:**
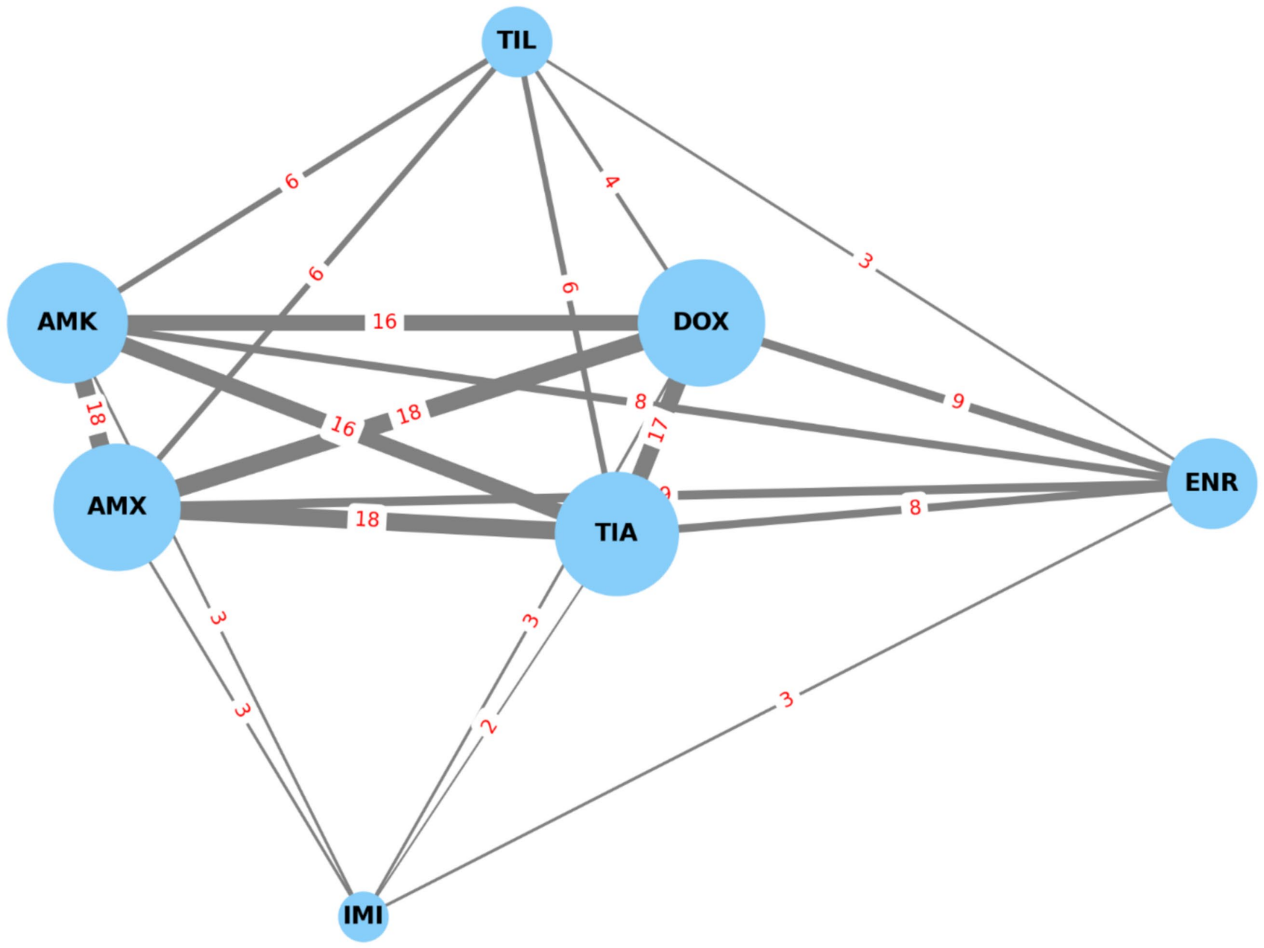
Resistance-based network graph of *Staphylococcus aureus* strains (*n* = 22) isolated from chickens in the Észak-Alföld region. The strongest associations were observed between enrofloxacin and potentiated sulphonamide, as well as between enrofloxacin and tiamulin. AMX, amoxicillin; AMK, amoxicillin-clavulanic acid; DOX, doxycycline; TIL, tylosin; TIA, tiamulin; ENR, enrofloxacin; PSA, potentiated sulphonamide (trimethoprim-sulfamethoxazole, 1:19).

Network analysis (Figure 4) visualized co-occurrence patterns of resistance, with frequent pairings including amoxicillin–doxycycline (18), amoxicillin–tiamulin (18), amoxicillin–clavulanic acid–doxycycline (16), and amoxicillin–clavulanic acid–tiamulin (16).

**Figure 5:**
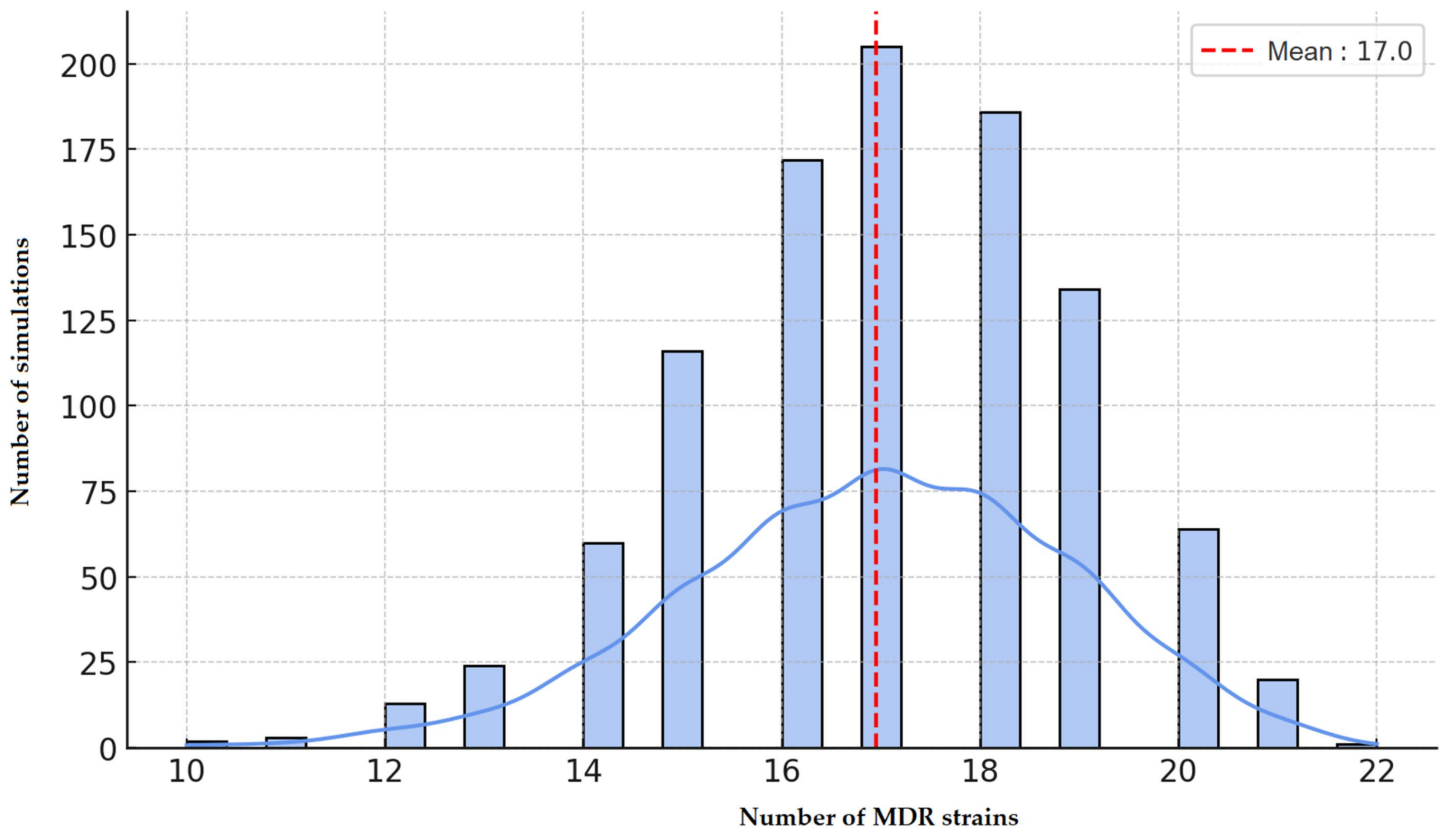
Stochastic estimation of the occurrence of multidrug-resistant (MDR) *Staphylococcus aureus* strains isolated from chickens in the Észak-Alföld region using Monte Carlo simulation. The mean number of multidrug-resistance (MDR) isolates was 17.0 over 10,000 iterations.

Monte Carlo simulations predicted an average of 17.0 MDR isolates per run (Figure 5), serving as a baseline to evaluate whether observed MDR frequencies exceeded those expected by chance.

**Figure 6:**
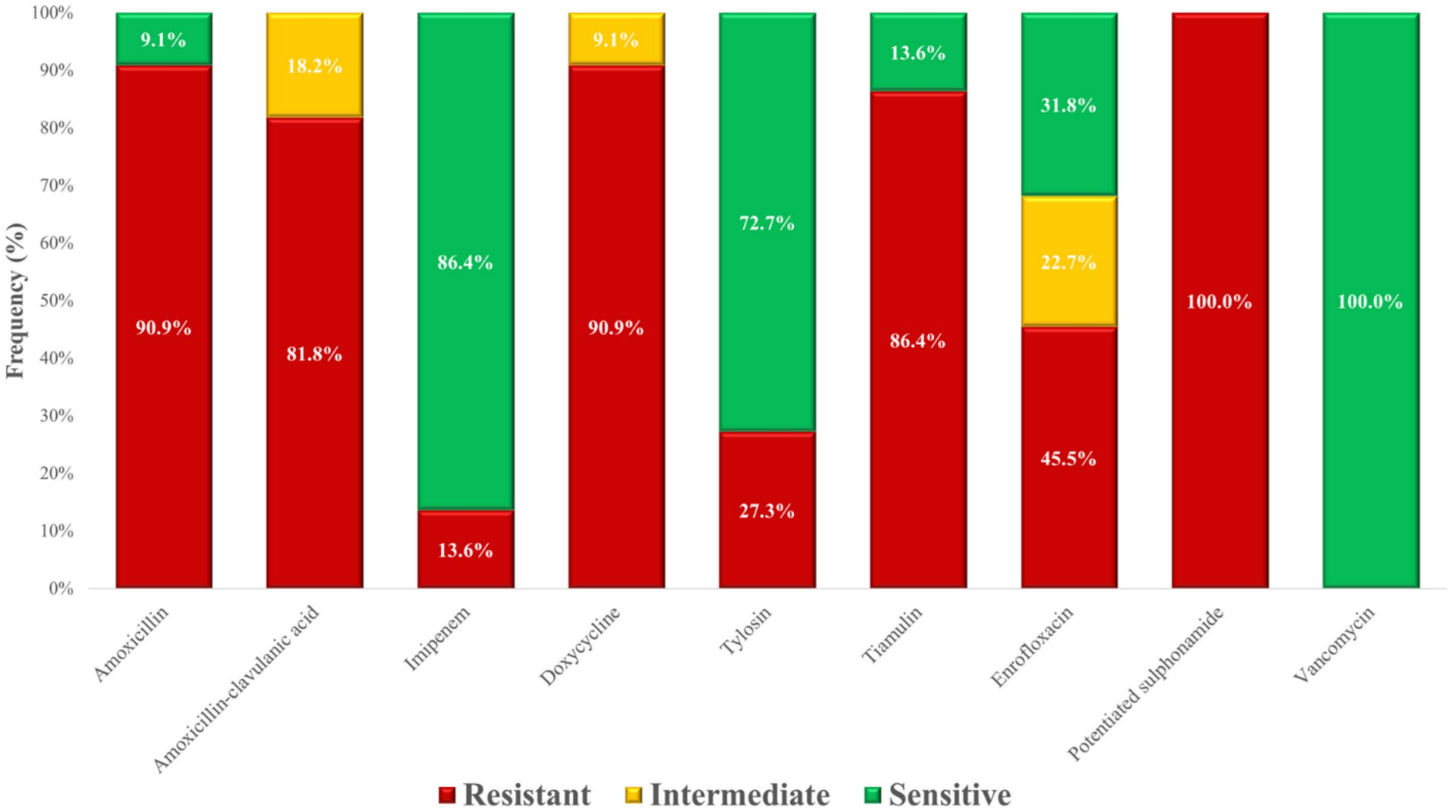
Antimicrobial resistance profile of *Staphylococcus aureus* strains (*n* = 22) isolated from chickens in the Észak-Alföld region.

MIC frequency distributions were compiled (Supplementary Table 1), and resistant versus susceptible proportions were plotted graphically (Figure 6). Complete resistance (100%) was recorded for potentiated sulphonamides. Resistance to amoxicillin was extremely high (90.9%), while the slightly lower rate observed for amoxicillin–clavulanic acid (81.8%). Particularly concerning was the 45.5% resistance to enrofloxacin, a critically important antimicrobial. By contrast, vancomycin, reserved as a last-resort agent for human medicine, retained full efficacy across all isolates.

### 3.2 *Enterococcus* isolates

**Figure 7:**
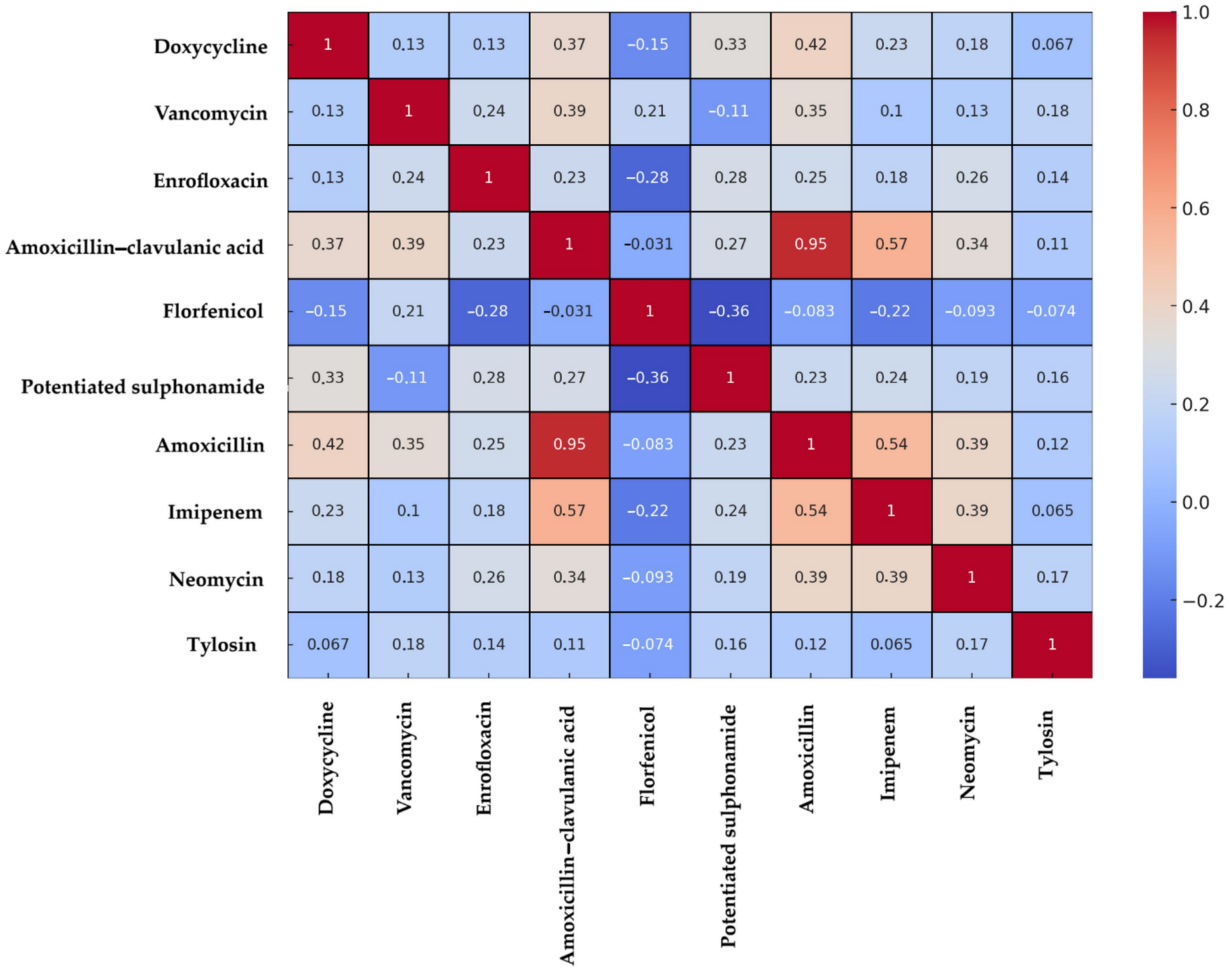
Spearman correlation heatmap of *Enterococcus* strains (*n* = 73) isolated from chickens in the Észak-Alföld region. Positive correlation values indicate co-occurrence of resistance to both agents, values near zero suggest independence, and negative correlations imply inverse associations.

The Spearman correlation heatmap (Figure 7) revealed several significant positive associations, most notably between amoxicillin and amoxicillin–clavulanic acid (0.95), amoxicillin–clavulanic acid and imipenem (0.57), and amoxicillin and imipenem (0.54).

**Figure 8:**
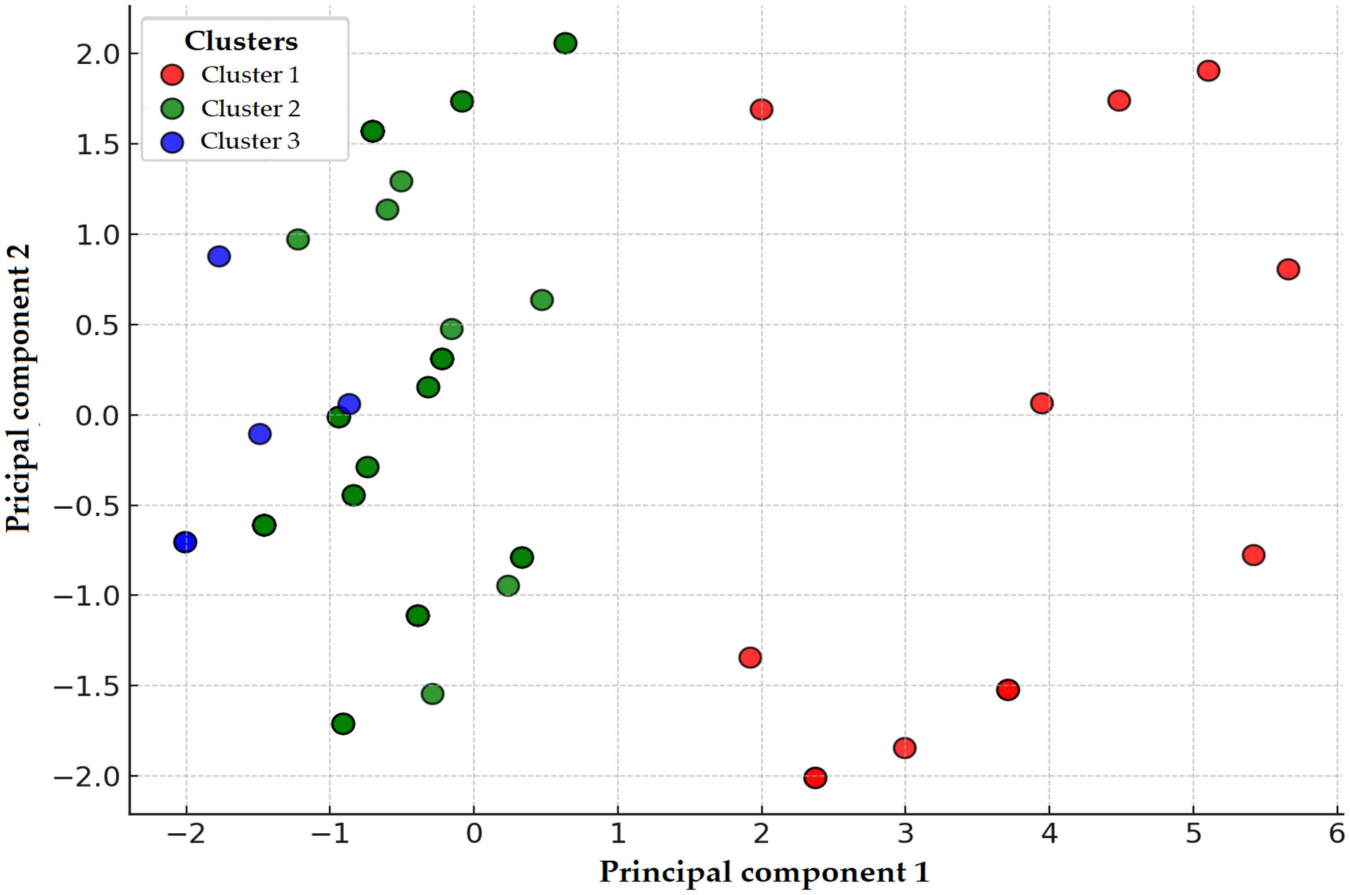
Principal component analysis (PCA) and clustering of *Enterococcus* strains (*n* = 73) isolated from chickens in the Észak-Alföld region. Red, green, and blue colors represent clusters 1, 2, and 3, respectively. Cluster 1 (red), characterized by extremely high-level resistance to florfenicol and tylosin; Cluster 2 (green), dominated by enrofloxacin resistance; and Cluster 3 (blue), showing elevated resistance to potentiated sulphonamide.

PCA combined with K-means clustering (Figure 8) delineated three distinct groups. Cluster 1 (red) predominantly comprised isolates with high MDR status, whereas the remaining clusters reflected lower resistance levels.

**Figure 9:**
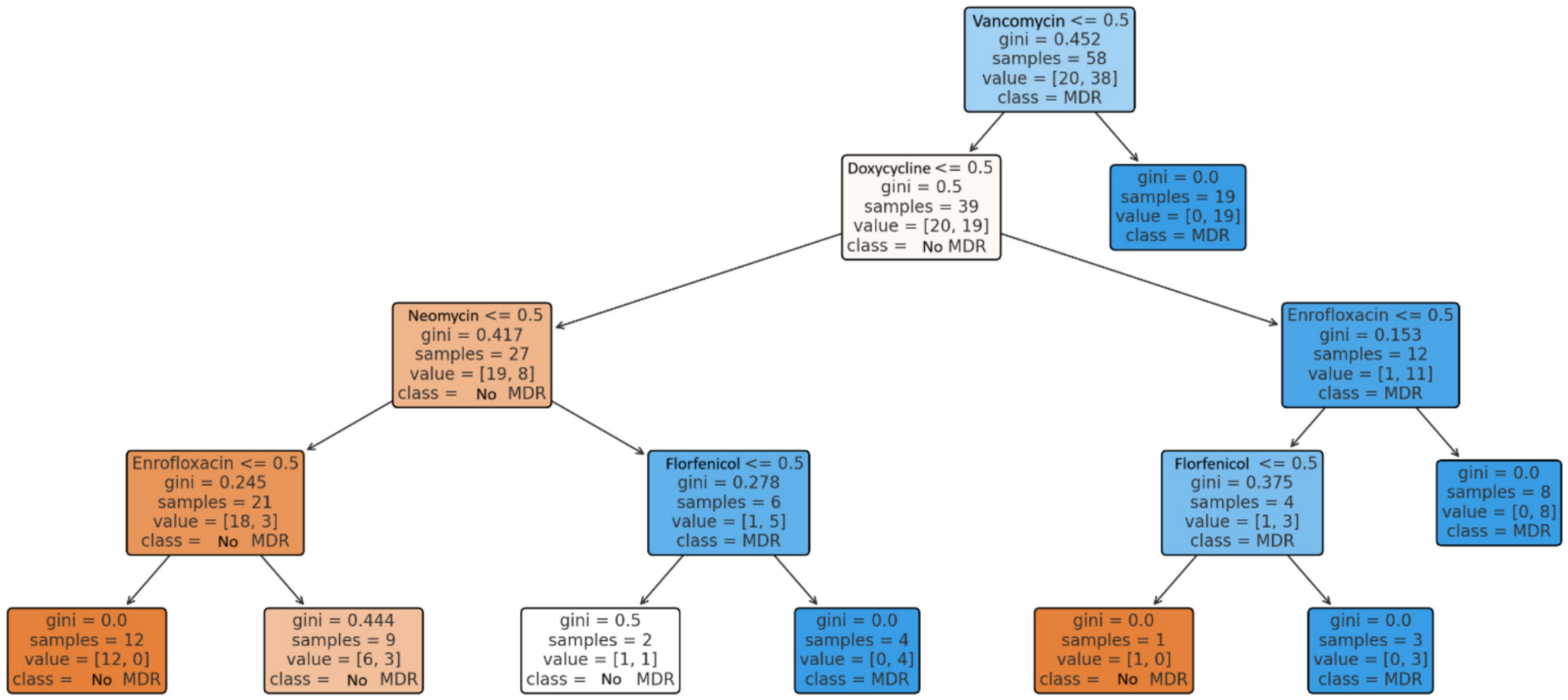
Prediction of multidrug-resistant (MDR) status of *Enterococcus* strains (*n* = 73) from the Észak-Alföld region using a decision tree model. The most relevant predictors were resistance to vancomycin, doxycycline and enrofloxacin.

The decision tree model (Figure 9) identified vancomycin, doxycycline, neomycin, and enrofloxacin as the strongest predictors of MDR. For example, isolates resistant to both vancomycin and doxycycline were highly likely also to exhibit resistance to neomycin or enrofloxacin, indicating strong combinatorial predictive power.

**Figure 10:**
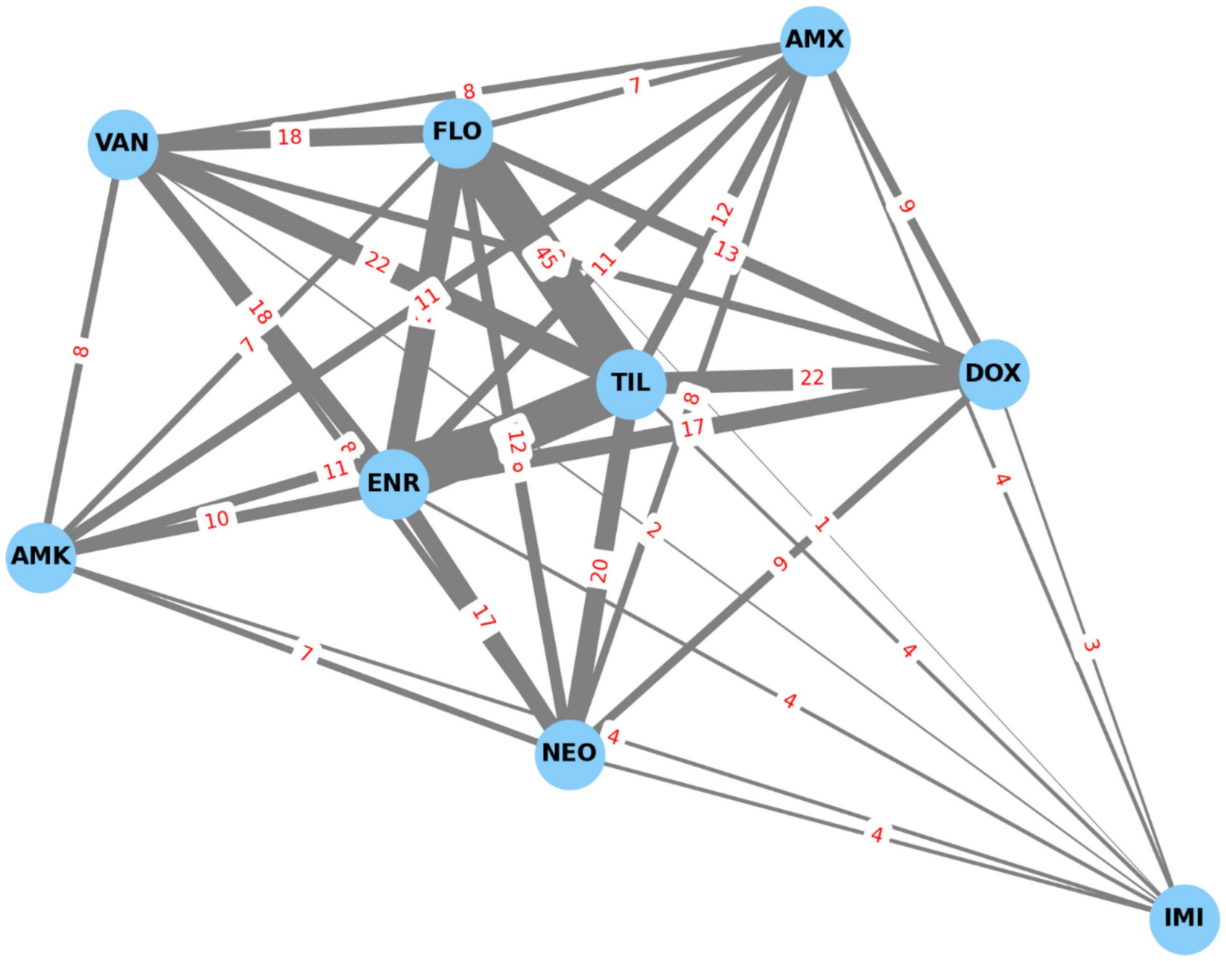
Resistance-based network graph of *Enterococcus* strains (*n* = 73) isolated from chickens in the Észak-Alföld region. The strongest associations were observed among florfenicol-tylosin and enrofloxacin-tylosin. AMX, amoxicillin; AMK, amoxicillin-clavulanic acid; NEO, neomycin; DOX, doxycycline; FLO, florfenicol; TIL, tylosin; ENR, enrofloxacin; VAN, vancomycin.

**Figure 11:**
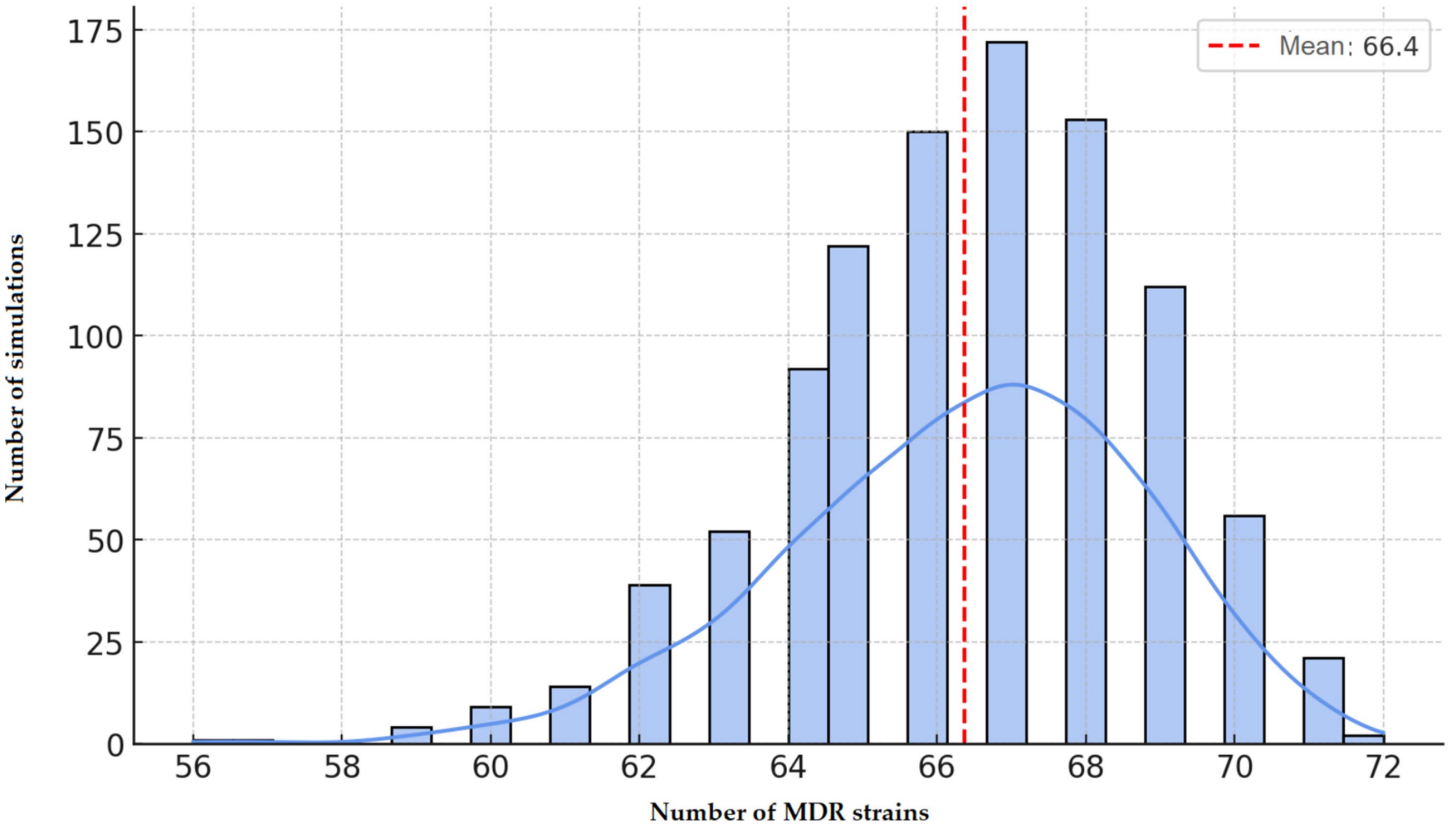
Monte Carlo-based stochastic estimation of multidrug-resistant (MDR) *Enterococcus* strains isolated from chickens in the Észak-Alföld region.

Network visualization (Figure 10) highlighted the most frequent co-occurring resistance pairing as florfenicol–tylosin (37). Simulation modeling estimated an average of 66.4 MDR isolates if resistance were randomly distributed. In reality, 47 MDR isolates were observed (Figure 11), slightly below the random expectation.

**Figure 12:**
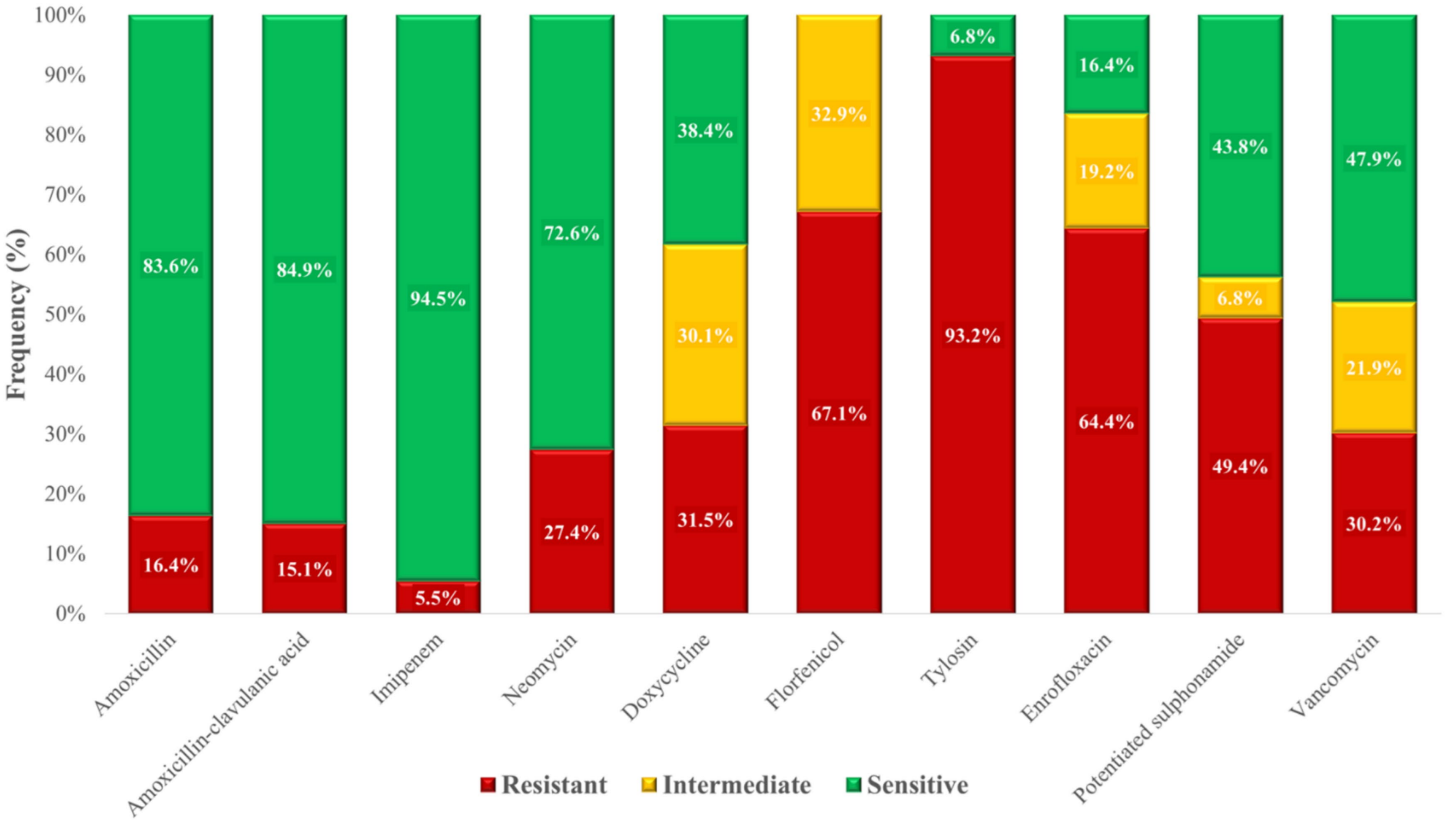
Antimicrobial resistance profile of *Enterococcus* strains (*n* = 73) isolated from chickens in the Észak-Alföld region.

Frequency tables (Supplementary Table 2) and graphical summaries (Figure 12) indicated the highest resistance rates to tylosin (93.2%). While resistance to imipenem, reserved exclusively as a public health safeguard, was low (5.5%), vancomycin resistance (30.2%) was worrisome. A majority of isolates (83.6%) were susceptible to amoxicillin.

### 3.3 *Escherichia coli* isolates

**Figure 13:**
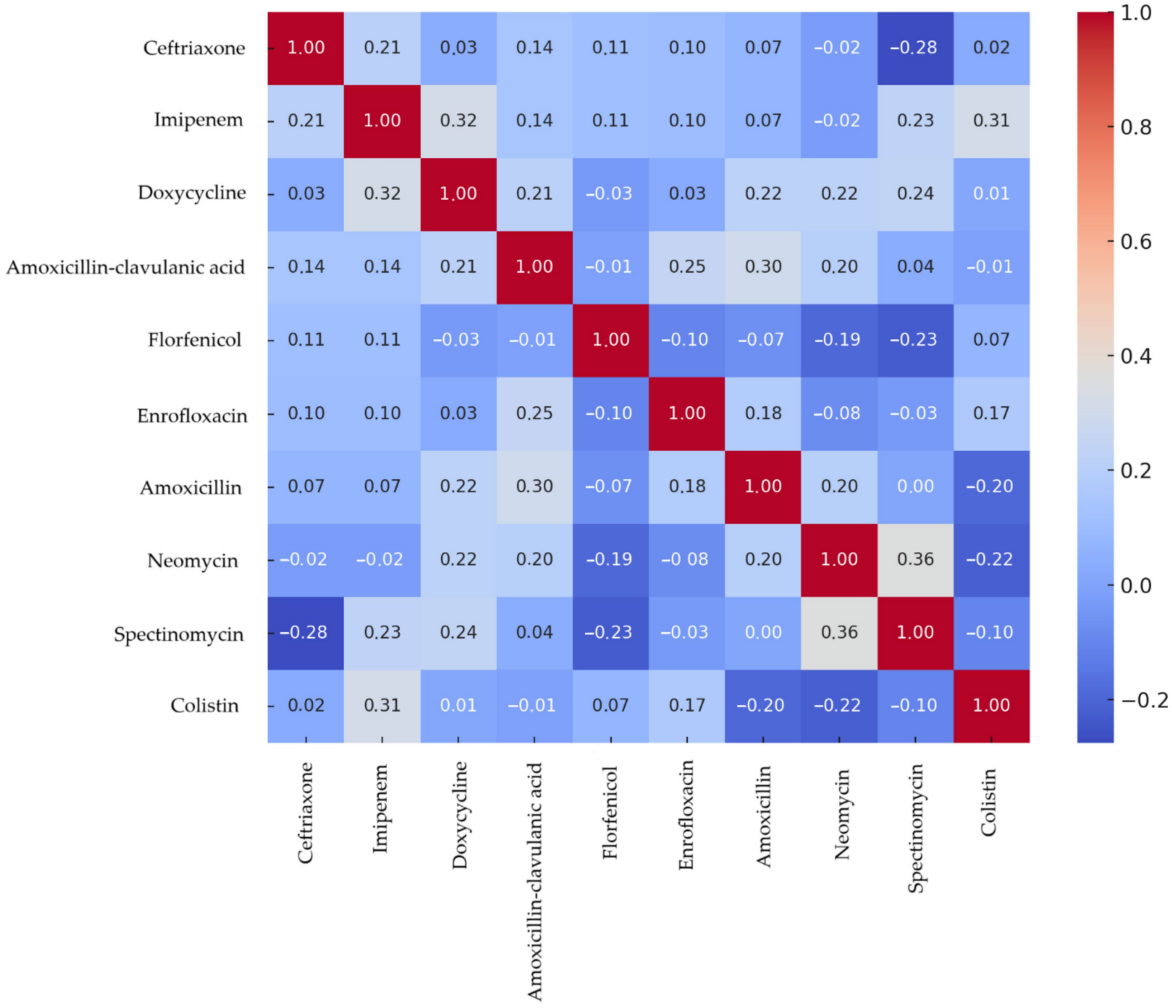
Spearman correlation heatmap of *Escherichia coli* strains (*n* = 71) isolated from chickens in the Észak-Alföld region. Positive correlations indicate co-resistance, values near zero indicate independence, and negative values suggest inverse resistance patterns.

Correlation analysis (Figure 13) demonstrated positive associations between multiple antimicrobials, with the strongest being neomycin–spectinomycin (0.36), imipenem–doxycycline (0.32), and imipenem–colistin (0.31).

**Figure 14:**
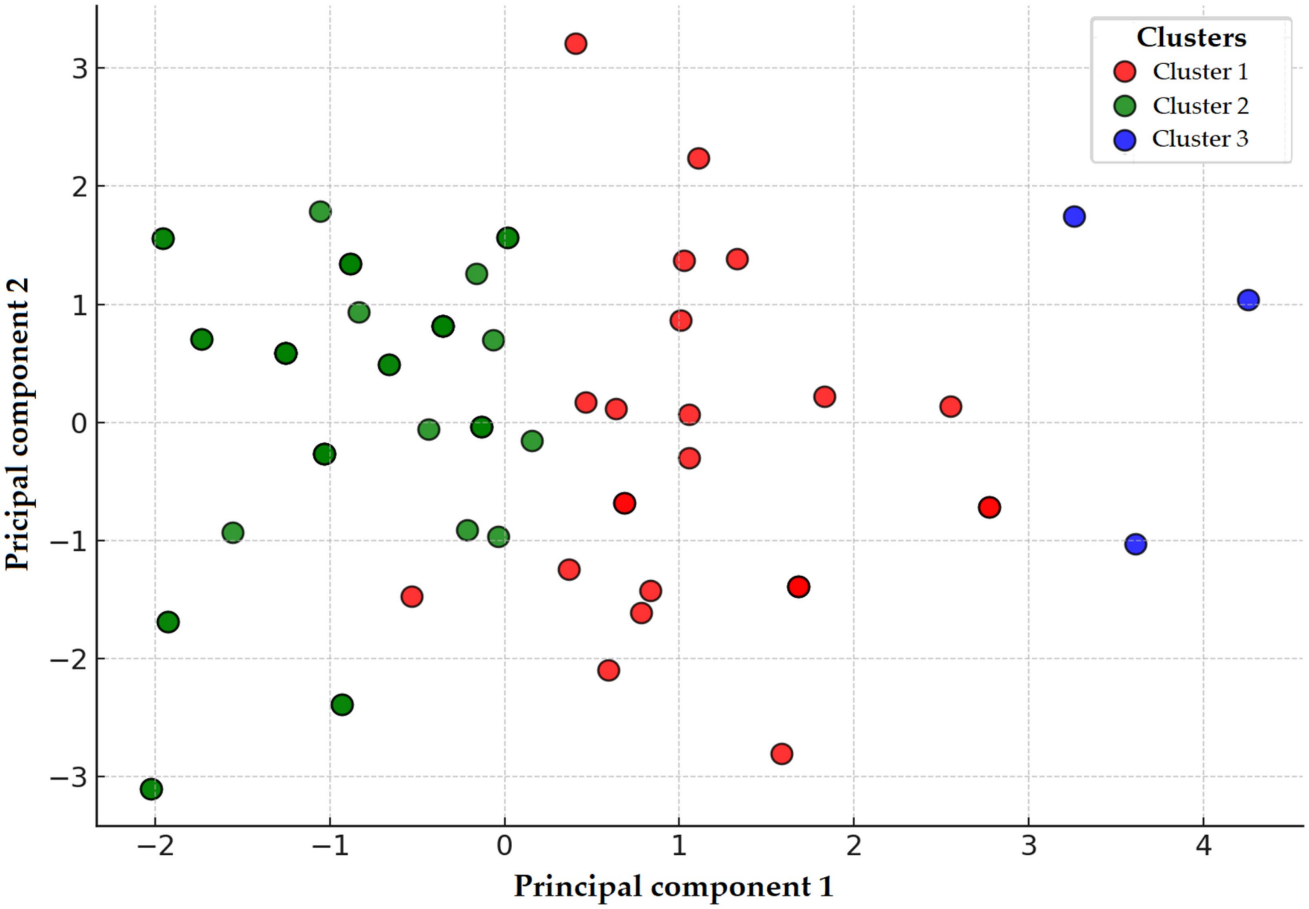
Principal component analysis (PCA) and clustering of *Escherichia coli* strains (*n* = 71) isolated from chickens in the Észak-Alföld region. Clusters 1, 2, and 3 are represented by red, green, and blue colors, respectively. Cluster 1 characterized by extremely high-level resistance to florfenicol and enrofloxacin; Cluster 2 dominated by neomycin and spectinomycin resistance; and Cluster 3 showing elevated resistance to amoxicillin and amoxicillin–clavulanic acid.

PCA and K-means clustering identified three distinct groups (Figure 14), even though all isolates were MDR. This observation suggests that heterogeneous resistance patterns persist within the MDR category, possibly reflecting genetic diversity or distinct selection pressures.

**Figure 15:**
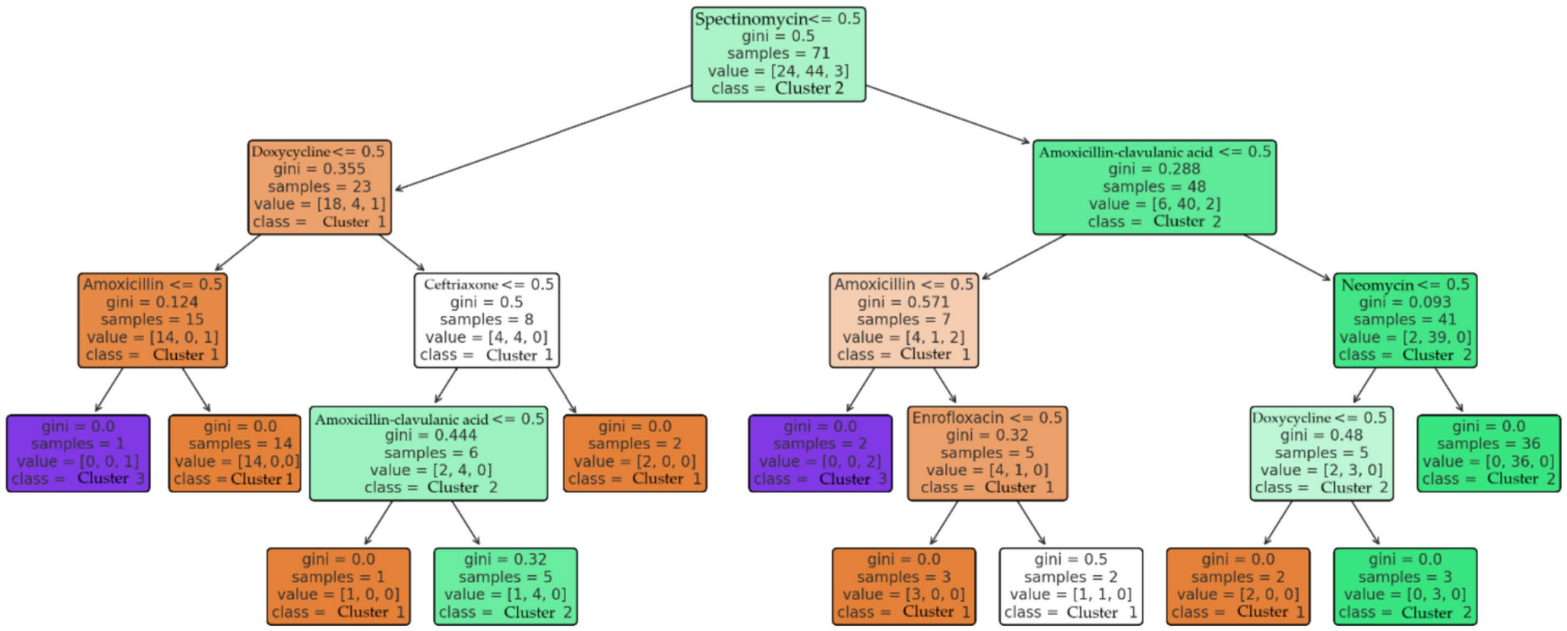
Prediction of multidrug-resistant (MDR) status of *Escherichia coli* strains (*n* = 71) from the Észak-Alföld region using a decision tree model. The most significant resistance traits were linked to spectinomycin, doxycycline, and amoxicillin-clavulanic acid.

Decision tree analysis (Figure 15) therefore focused on distinguishing among clusters rather than MDR status itself. Cluster differentiation was primarily driven by resistance to spectinomycin, doxycycline, and amoxicillin–clavulanic acid.

**Figure 16:**
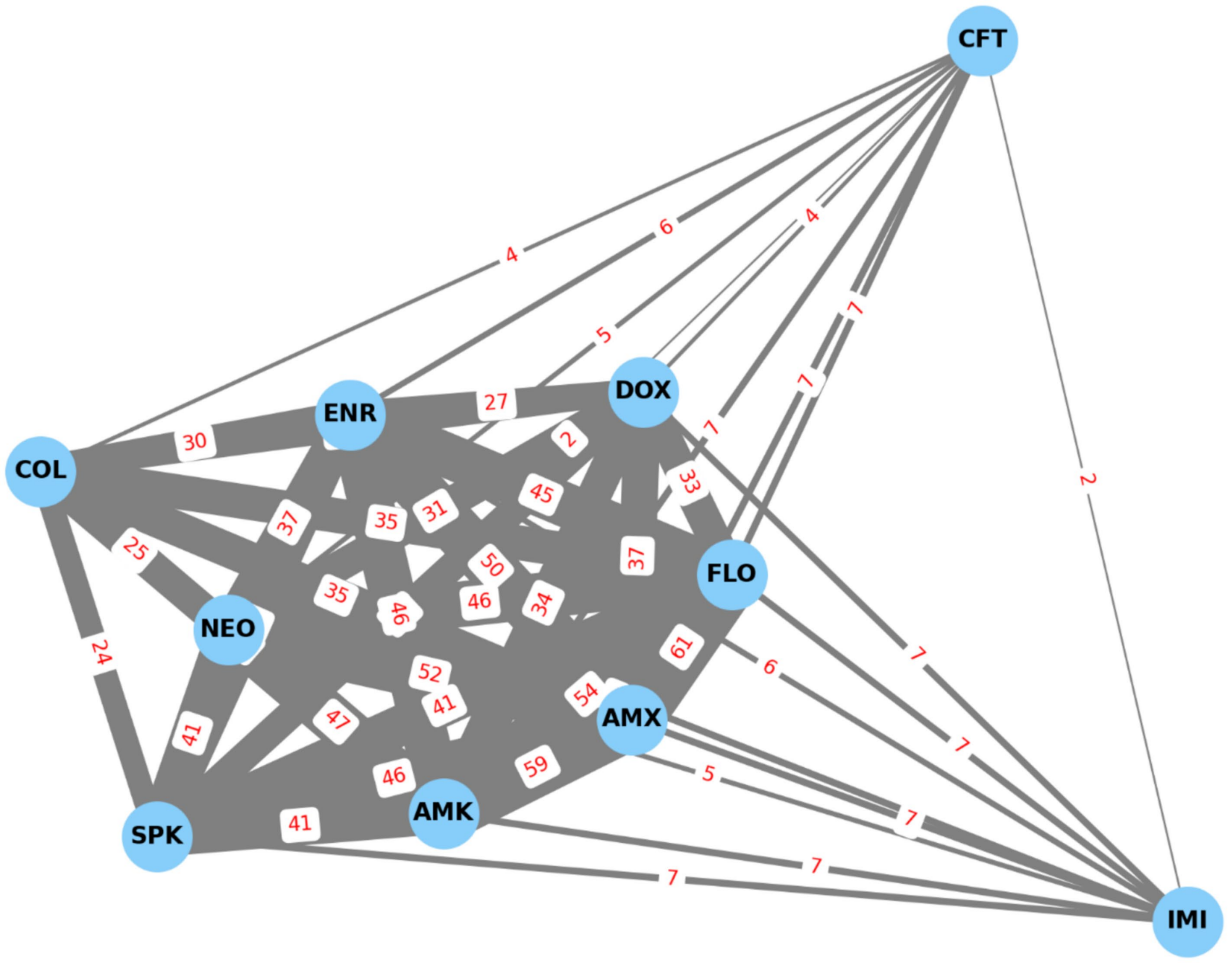
Resistance-based network graph of *Escherichia coli* strains (*n* = 71) isolated from chickens in the Észak-Alföld region. The strongest associations were found among amoxicillin-neomycin, amoxicillin-amoxicillin-clavulanic acid and amoxicillin-florfenicol. AMX, amoxicillin; AMK, amoxicillin-clavulanic acid; NEO, neomycin; DOX, doxycycline; FLO, florfenicol; CFT, ceftriaxone; ENR, enrofloxacin; SPK, spectinomycin; COL, colistin; IMI, imipenem.

Network mapping (Figure 16) revealed the strongest co-resistance relationships between amoxicillin–amoxicillin–clavulanic acid (38), amoxicillin–florfenicol (39), and amoxicillin–neomycin (40), highlighting potential shared resistance mechanisms.

**Figure 17:**
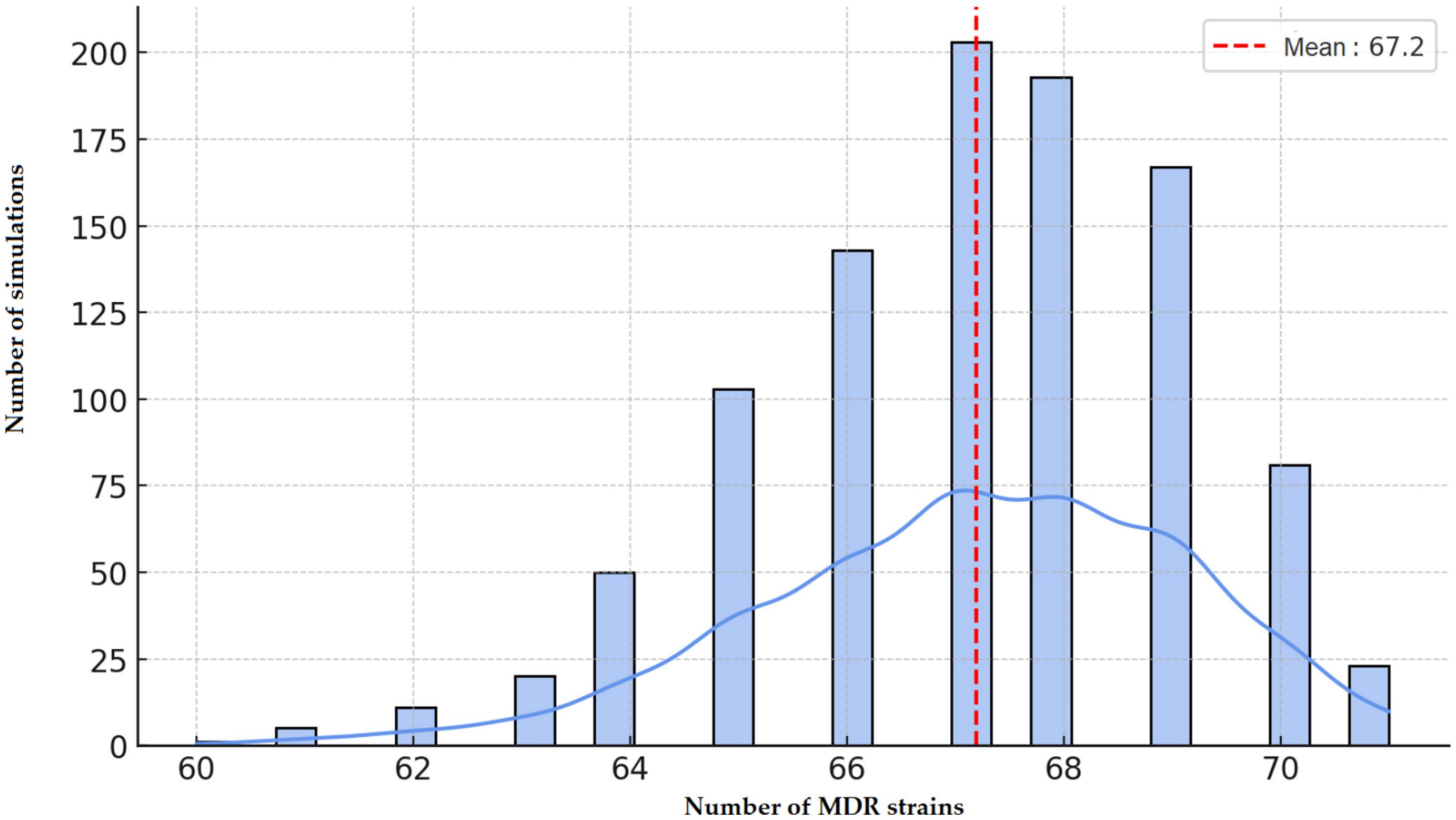
Monte Carlo simulation-based stochastic estimation of the occurrence of multidrug-resistant (MDR) *Escherichia coli* strains in the Észak-Alföld region.

Monte Carlo simulations predicted an average of 67.2 MDR isolates if resistance were randomly distributed (Figure 17). The observed value of 71 isolates exceeded this baseline, strongly supporting the presence of direct selection pressure in the region.

**Figure 18:**
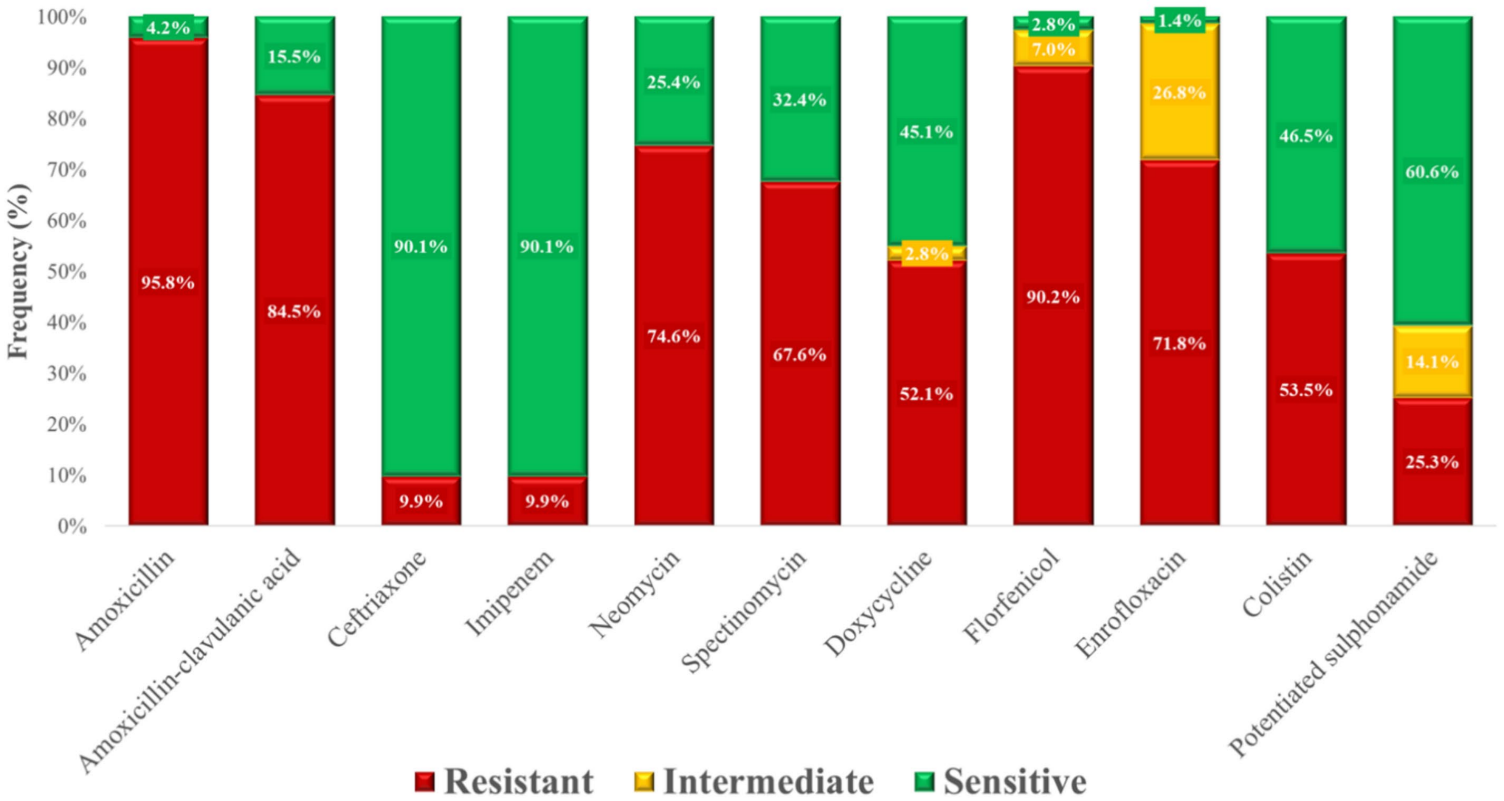
Antimicrobial resistance profile of *Escherichia coli* strains (*n* = 71) isolated from chickens in the Észak-Alföld region.

Finally, MIC frequency tables (Supplementary Table 3) and susceptibility proportions (Figure 18) demonstrated the highest resistance rates against amoxicillin (95.8%), followed by florfenicol (90.2%).

### 3.4 Multidrug-(MDR), extensively drug (XDR), and pandrug resistant (PDR) isolates

Resistance phenotypes were classified according to internationally accepted definitions. Isolates were considered MDR if they showed non-susceptibility to at least one agent in three or more antimicrobial categories. Extensively drug-resistant (XDR) isolates were defined as those non-susceptible to all but one or two categories, while pandrug-resistant (PDR) isolates exhibited non-susceptibility to all agents across all antimicrobial categories tested. These definitions were applied uniformly across bacterial species to ensure consistency in resistance categorization (41).

**Figure 19:**
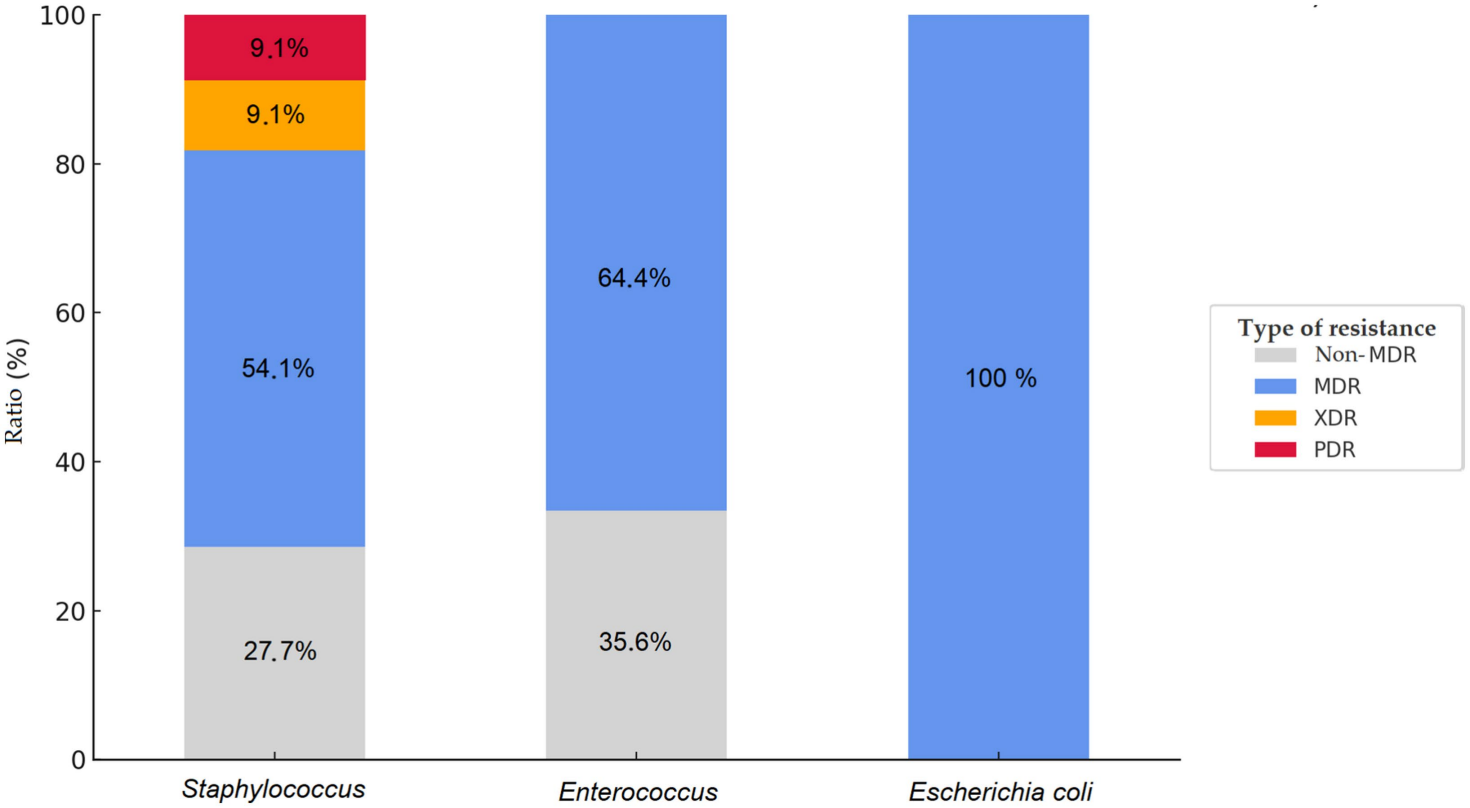
Comparative distribution of multidrug-resistant (MDR), extensively drug-resistant (XDR), and pandrug-resistant (PDR) strains among isolates from the Észak-Alföld region.

Comparative analysis of the Észak-Alföld region revealed notable differences in the resistance profiles of the three bacterial species investigated—*S. aureus*, *Enterococcus*, and *E. coli* (Figure 19). Among *E. coli* isolates, all strains were classified as MDR, corresponding to a prevalence of 100%, while no XDR or PDR isolates were identified. In *Enterococcus*, 64.4% of isolates were MDR, again without evidence of XDR or PDR status. By contrast, *S. aureus* isolates displayed greater variability: 54.1% were MDR, while two isolates were classified as XDR and two as PDR. Thus, although *S. aureus* showed a lower overall MDR rate compared to *E. coli*, it was the only species in which isolates with extensive and complete resistance were detected.

### 3.5 Comparison with regional human resistance data

**Figure 20:**
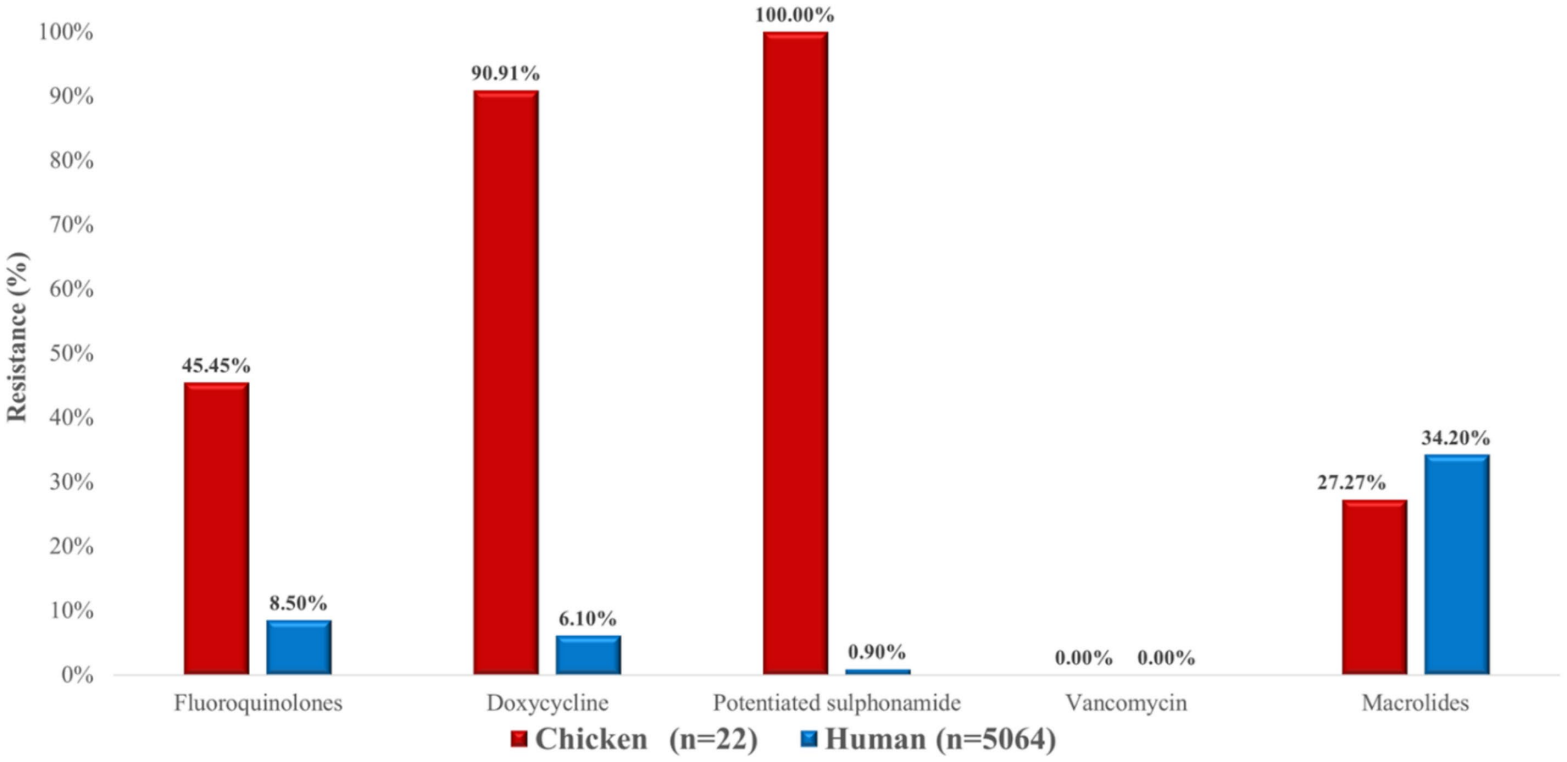
Comparison of antibiotic resistance between *Staphylococcus aureus* bacterial isolates from chickens (*n* = 22) and human sources (*n* = 5,064) across different antimicrobial classes. Notably higher resistance rates were observed in poultry-derived strains against potentiated sulphonamides, fluoroquinolones, and doxycycline.

The resistance levels of *S. aureus* isolates from poultry were generally higher than those observed in human samples for most antimicrobial classes (Figure 20). Striking differences were found for potentiated sulphonamides (100.0% vs. 0.9%) and tetracyclines (90.9% vs. 6.1%). In contrast, macrolide resistance was comparable between poultry and human isolates (approximately 27–34%). No resistance to vancomycin was detected in either group.

**Figure 21:**
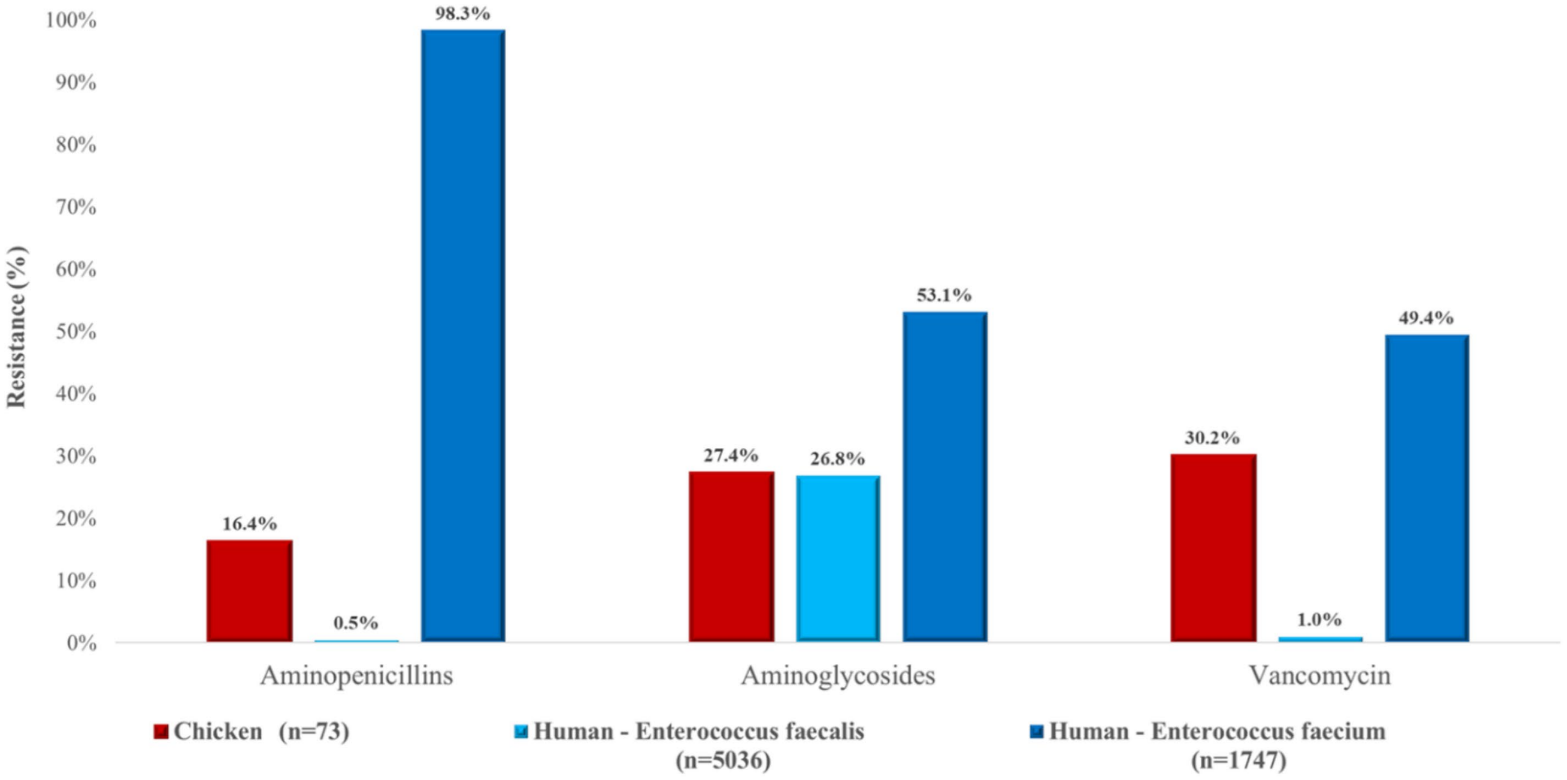
Comparison of antibiotic resistance in *Enterococcus* isolates from chickens (*n* = 73) and human sources, including *Enterococcus faecalis* (*n* = 5,036) and *Enterococcus faecium* (*n* = 1,747). Notably, resistance to aminopenicillins was extremely high in *E. faecium*, while poultry-derived isolates showed moderate resistance across all tested antimicrobial classes.

For *Enterococcus*, a more heterogeneous pattern emerged (Figure 21). Resistance to aminopenicillins in poultry-derived isolates was 16.4%, compared with 0.5% in human *E. faecalis* isolates but an extremely high 98.3% in *E. faecium*. Resistance to aminoglycosides was similar in poultry (27.4%) and human *E. faecalis* isolates (26.8%), while higher rates were observed in human *E. faecium* (53.1%). Vancomycin resistance was 30.1% in poultry isolates, lower than the 49.4% observed in human *E. faecium*.

**Figure 22:**
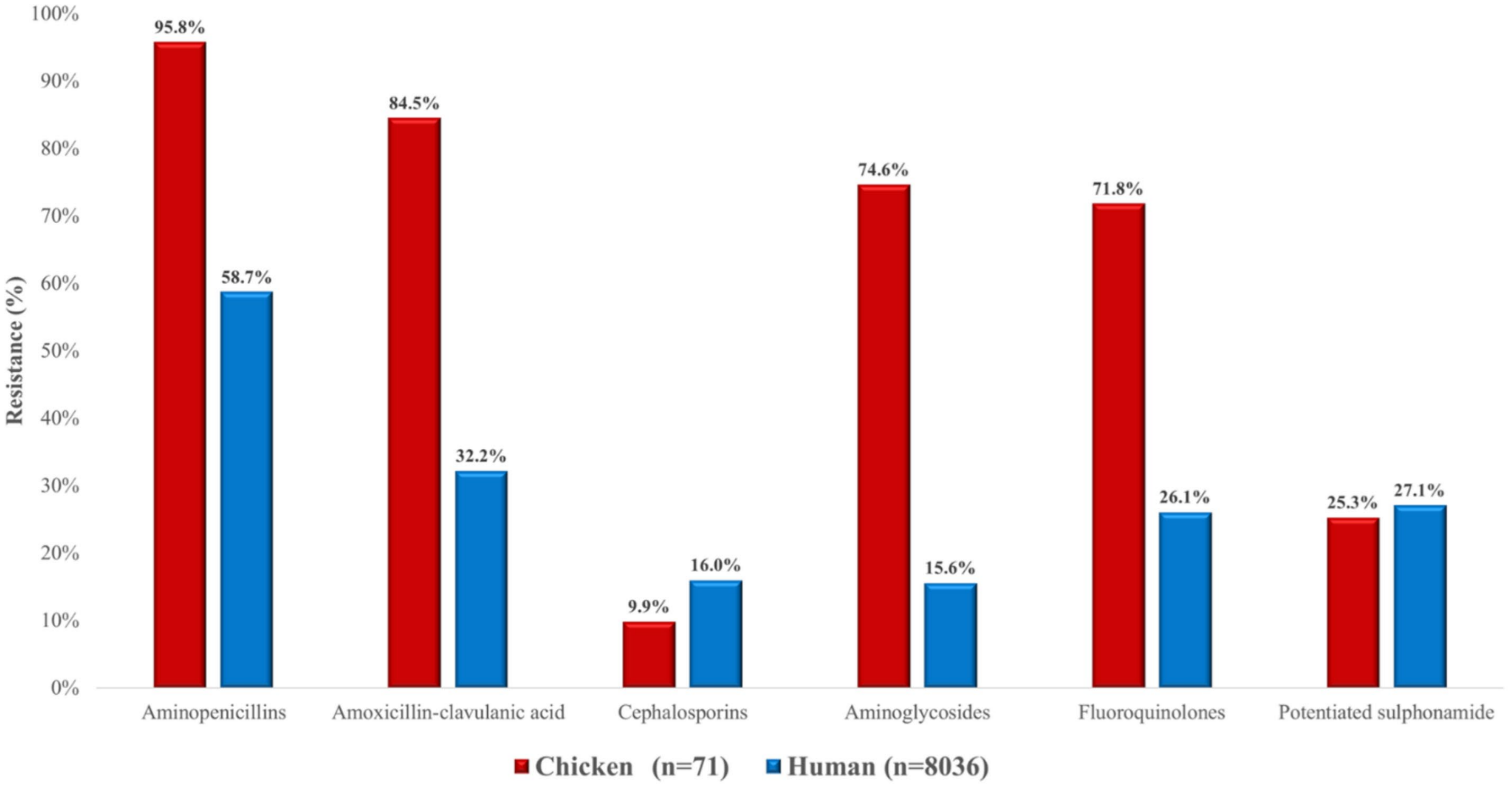
Comparison of antibiotic resistance in *Escherichia coli* isolates from chickens (*n* = 71) and human sources (*n* = 8,036) across various antimicrobial classes. Poultry-derived strains exhibited higher resistance in most categories, especially aminoglycosides, fluoroquinolones, and potentiated sulphonamides.

For *E. coli*, poultry-derived isolates demonstrated markedly higher resistance than human isolates across several antimicrobial classes (Figure 22). Aminoglycoside resistance was especially pronounced in poultry strains (74.6% vs. 15.6%), as were differences for fluoroquinolones (71.8% vs. 26.1%) and amoxicillin–clavulanic acid (84.5% vs. 32.2%). In contrast, resistance to potentiated sulphonamides was comparable (25.4% vs. 27.1%). Interestingly, resistance to cephalosporins was lower in poultry isolates (9.9%) compared with human isolates (16.0%).

## 4 Discussion

This study investigated antimicrobial resistance in 166 bacterial isolates obtained from poultry in the Észak-Alföld (North Great Plain) region of Hungary. The overall prevalence of MDR strains was high, and notably, both XDR and PDR isolates were identified. It should be noted that the sampling was not part of a disease investigation but of a surveillance-oriented program in clinically healthy flocks. While clinical health was confirmed by the absence of visible symptoms at the time of sampling, no laboratory testing (e.g., serology or postmortem diagnostics) was performed to confirm subclinical status. Therefore, the findings should be interpreted within the context of non-diseased, field-level conditions. Although species identification was confirmed using MALDI-TOF mass spectrometry, molecular methods (e.g., sequencing) were not applied, which may limit the resolution for closely related strains.

Among the 22 *S. aureus* isolates analyzed, resistance to amoxicillin was 90.9%, while resistance to amoxicillin–clavulanic acid reached 81.8%. This high resistance to both the penicillin and its β-lactamase inhibitor combination suggests that resistance mechanisms beyond classical β-lactamase production may be involved, such as penicillin-binding protein (PBP) alterations, including those mediated by *mecA* or *mecC*. As β-lactamase activity was not directly assessed in this study, no definitive conclusions can be drawn regarding its role. In comparison, Kim et al. (41), reported a much lower resistance rate of 51.2% to penicillins, suggesting that differences in antibiotic use and selective pressure may explain regional variation. Notably, neither amoxicillin–clavulanic acid nor imipenem are licensed for use in poultry, but both have public health relevance as they represent critical drugs for human medicine.

Resistance to doxycycline was alarmingly extremely high (90.9%), exceeding the values reported by Miranda et al. (58.4%) (42) and Kim et al. (38.8% to tetracyclines) (41). Similarly, enrofloxacin resistance reached 45.5%, higher than the 33.9% resistance to ciprofloxacin reported by Kim et al. (41). These differences likely reflect varying antibiotic usage patterns, though our findings are limited by the relatively small sample size of *S. aureus* isolates, which may reduce representativeness. Encouragingly, all isolates remained susceptible to vancomycin, which is strictly reserved as a last-line drug in human healthcare. However, Mkize et al. (43) reported vancomycin resistance in 14% of isolates from farm fecal samples and up to 61.9% in slaughterhouse isolates. This discrepancy may be explained by cross-resistance or the historical use of avoparcin, a glycopeptide previously applied as a growth promoter in certain countries, including South Africa (44).

The extremely high resistance rates observed against doxycycline, amoxicillin, tiamulin, and potentiated sulphonamides are particularly concerning, as these are frequently used in poultry production. PCA and clustering analysis suggested distinct resistance patterns, potentially reflecting either genetically diverse mechanisms or different exposure environments. The decision tree analysis identified specific drug combinations predictive of MDR status, while the resistance network highlighted strong associations such as amoxicillin–doxycycline and amoxicillin–tiamulin, suggestive of potential cross-resistance (45). Monte Carlo simulations confirmed that the observed MDR prevalence exceeded random expectation, providing further evidence of selective antimicrobial pressure in this region. Collectively, these results highlight the urgent need to preserve the efficacy of medically important antibiotics by restricting their use in poultry farming and promoting rational, evidence-based treatment strategies.

A total of 73 *Enterococcus* isolates were analyzed. Resistance to amoxicillin was moderate (16.4%), which is encouraging given that penicillins remain first-line drugs for enterococcal infections. By contrast, Bekele et al. (46) reported higher resistance rates (43–63%), while Lemsaddek et al. (47) found marked differences depending on production system, with only 6.25% resistance under free-range conditions but 33.3% under industrial farming. These observations suggest that production practices, stress, and associated antibiotic use can exert strong selective pressure. Resistance to amoxicillin–clavulanic acid was 15.1%, with highly variable values reported elsewhere (Pesavento et al., 1.47% (48), Ayeni et al., 73.3% (37)). Since *Enterococcus* does not produce β-lactamase, such variability likely reflects differences in antibiotic usage patterns across regions.

Resistance to neomycin was 27.4%, considerably lower than the 83% reported by Lanza et al. (49). Doxycycline resistance was 31.5%, with an additional 30.1% of isolates showing intermediate susceptibility. These results are consistent with previous reports ranging from 26 to 70% depending on production system (46, 49, 50). Both neomycin and doxycycline are widely used in poultry, and the observed variability is most likely caused by differences in the intensity and patterns of their use across farms and production systems, which in turn generate varying levels of selective pressure. Florfenicol resistance was remarkably very high (67.1%). This may be linked to its extensive use in poultry farming and the dissemination of resistance genes such as *fexA* and *floR*, or efflux pump genes (e.g., *efrAB*), which can spread via horizontal gene transfer within the gut microbiome. The strong biofilm-forming capacity of *Enterococcus* further enhances persistence of such traits (51). In contrast to our findings, Schwaiger et al. (63) reported complete susceptibility, while Karunarathna et al. (64) and Kim et al. (40) described very low and moderate resistance rates (0.4–18.7%). These discrepancies may be explained by regional usage practices, differences in feed composition, microbiome complexity, or taxonomic composition of isolates.

Resistance to tylosin was extremely high (93.2%), exceeding even the 53.0–63.6% reported by Kim et al. (40). This may reflect the widespread and long-standing use of tylosin and other macrolides in poultry production in the region, which can exert selective pressure and promote cross-resistance within this antibiotic class. Enrofloxacin resistance (64.4%) was also concerning, though comparable to the 83.3% observed by Oliveira et al. (65), However, other studies have reported much lower resistance rates (5.1–29.6%) (39, 51) again reflecting regional differences in fluoroquinolone usage. Resistance to potentiated sulphonamides (trimethoprim–sulfamethoxazole) was 49.4%, higher than the 32.9–36.6% reported by Makarov et al. (39) but much higher values were observed elsewhere, ranging from 65% to over 90% (39, 47, 52). The variability underscores the role of local antibiotic use in shaping resistance. Particularly concerning was the detection of vancomycin resistance in 30.1% of isolates. While several studies reported no resistance (47, 49, 51), others documented values up to 92% (37, 46, 51). The emergence of vancomycin-resistant enterococci (VRE) is a serious public health threat, likely linked to historical avoparcin use as a growth promoter (44).

Overall, the 64.4% prevalence of MDR among *Enterococcus* isolates in this region is substantial. Clustering and decision tree analysis highlighted vancomycin and doxycycline resistance as key drivers of MDR status. The network and simulation analyses further supported that resistance patterns are not random, but rather shaped by directed selection pressure, reflecting the impact of antimicrobial use in poultry production. These findings emphasize the importance of antibiotic stewardship and the need for a One Health perspective when addressing resistance in *Enterococcus*.

We examined 71 *E. coli* isolates, all of which were MDR. Resistance to amoxicillin was 95.8%, with similarly extremely high resistance to amoxicillin–clavulanic acid (84.5%). While some studies have reported much lower resistance to ampicillin (19–65%) (38, 53–56), others described similarly very high values (38, 54). Such variability may reflect regional antimicrobial usage patterns and the known diversity of resistance mechanisms in *E. coli*, including—but not limited to—β-lactamase and extended-spectrum β-lactamase (ESBL) production. However, as this study did not perform phenotypic or genotypic confirmation of β-lactamase activity, these mechanisms remain hypothetical in the current context. Resistance to ceftriaxone and imipenem was relatively low (9.9%), although other studies found higher rates, particularly for third-generation cephalosporins (38, 54, 57). These results nevertheless highlight the potential for dissemination of ESBL-producing *E. coli* in poultry populations.

Resistance to aminoglycosides was widespread, with neomycin resistance at 74.6% and spectinomycin resistance at 67.6%. These findings are consistent with very high levels of aminoglycoside resistance reported elsewhere (38, 58), though some studies noted much lower rates (38, 54, 59). Doxycycline resistance was 52.1%, again reflecting the strong selective pressure from tetracycline use in animal husbandry. Florfenicol resistance was particularly alarming (90.2%), in stark contrast to the very low or absent resistance described in other studies (60, 61). Similarly, enrofloxacin resistance (71.8%) was much higher than many published values (38, 53). Most concerning was the 53.5% resistance to colistin, a last-resort antibiotic in human medicine, compared to negligible resistance levels in other reports (60, 62, 66). This unexpectedly high resistance rate may be associated with the continued authorized use of colistin in food-producing animals, particularly for controlling enteric infections caused by *E. coli*. In Hungary, the use of colistin remains legally permitted under strict veterinary prescription, in line with Regulation (EU) 2019/6 and current national legislation. Although colistin use has been banned or restricted in several European countries, it is still authorized in others, including Hungary, primarily for treating gastrointestinal infections in livestock. Moreover, the presence and dissemination of plasmid-mediated colistin resistance genes (e.g., *mcr-1*), previously reported in *E. coli* from poultry, may contribute to the elevated resistance observed. These findings highlight the importance of monitoring the legal context of antimicrobial use and underscore the urgent need for molecular surveillance to clarify the underlying resistance mechanisms and inform policy decisions. This poses a major threat given the critical role of colistin in treating MDR Gram-negative infections in humans. Importantly, resistant bacteria originating from poultry can enter the food chain and be transmitted to humans, where they may cause hard-to-treat infections and significantly compromise therapeutic options.

Potentiated sulphonamide resistance (25.3%) was lower than in several other reports (38, 53), which may be explained by the declining use of these compounds after decades of overexploitation. The re-emergence of susceptible wild-type strains could account for this relative reduction. Finding that all *E. coli* isolates were MDR is deeply concerning. Correlation, clustering, and network analyses revealed frequent co-resistance patterns, especially involving amoxicillin–amoxicillin-clavulanic acid, amoxicillin–florfenicol, and amoxicillin–neomycin. Monte Carlo simulations confirmed that the high prevalence of MDR was not random but likely reflect strong region-specific selection pressure.

Our results demonstrate that bacteria from poultry in the Észak-Alföld region of Hungary harbor extremely high levels of antimicrobial resistance, including MDR, XDR, and even PDR phenotypes. Resistance levels frequently exceeded those reported in human clinical isolates, particularly for *E. coli* and *S. aureus*, raising serious public health concerns. The non-random clustering of resistance traits and the strong predictive value of certain antibiotics for MDR status further indicate that these patterns are driven by antimicrobial use practices. The observed similarity in resistance patterns across *E. coli*, *Enterococcus* spp., and *S. aureus* may reflect shared selection pressures in the production environment, such as the routine use of specific antibiotic classes at the flock level. Additionally, while these bacterial species differ in phylogeny and ecological niches, the potential role of horizontal gene transfer and common environmental reservoirs (e.g., litter, feed, or water systems) cannot be excluded.

Further studies incorporating whole-genome sequencing and farm-level antimicrobial usage data would be needed to clarify these associations.

This study reinforces the need for enhanced antimicrobial stewardship and surveillance in the poultry sector. Measures should include reduced and targeted use of critically important antimicrobials, promotion of alternative approaches (such as probiotics, plant extracts, and antimicrobial peptides), and harmonization of veterinary and human health policies under a One Health framework. Without decisive intervention, the continued spread of MDR, XDR, and PDR bacteria from poultry to humans may pose increasingly severe consequences for both animal and public health.

## 5 Conclusion

Our findings clearly underscore the critical importance of conducting regular, large-scale antimicrobial susceptibility testing. Such investigations provide essential insights into the resistance profiles of bacteria, particularly regarding MDR. While the majority of first-line antibiotics retained their effectiveness against *Enterococcus* spp., resistance to critically important antimicrobials for human health—especially among *E. coli* and *S. aureus* isolates—remains a cause for serious concern. The rising trends in antimicrobial resistance over recent years serve as a strong warning that a multidisciplinary approach is urgently needed, with closer collaboration between veterinary and public health professionals. Implementing the One Health concept must extend beyond theory into practice, encompassing preventive strategies, educational programs, and strengthened cross-sectoral communication. Only through such integrated efforts can the growing threat of resistance be effectively mitigated, thereby safeguarding human health in the long term.

## Data Availability

The original contributions presented in the study are included in the article/Supplementary material, further inquiries can be directed to the corresponding authors.
