# Supplementary material for: The prevalence of antimicrobial resistance of bacterial strains from large-scale healthy chicken flocks in the Észak-Alföld region of Hungary
Source: Front Vet Sci. 2026 Jan 12;12:1709725. doi: 10.3389/fvets.2025.1709725 (PMC12832369; doi:10.3389/fvets.2025.1709725)
Supplement: Supplementary file 1 [file Supplementary_file_1.docx]

Supplementary Material

**Supplementary Table 1** Frequency table of minimum inhibitory concentration (MIC) values for antimicrobial agents with established clinical breakpoints in *Staphylococcus* *aureus* isolates (*n*=22) from chickens in the Észak-Alföld region. The upper row for each compound displays the number of isolates, while the lower row shows the corresponding percentage. Vertical red lines indicate the clinical breakpoint, and green lines represent the epidemiological cutoff value (ECOFF).

| Antibiotics | ^1^Breakpoint | 0.001 | 0.002 | 0.004 | 0.008 | 0.016 | 0.031 | 0.063 | 0.125 | 0.25 | 0.5 | 1 | 2 | 4 | 8 | 16 | 32 | 64 | 128 | 256 | 512 | 1024 | MIC_50_ | MIC_90_ | ^2^ECOFF |
| --- | --- | --- | --- | --- | --- | --- | --- | --- | --- | --- | --- | --- | --- | --- | --- | --- | --- | --- | --- | --- | --- | --- | --- | --- | --- |
|  | µg/mL | | | | | | | | | | | | | | | | | | | | | | | | |
| Amoxicillin | 0.5 |  |  |  |  |  |  |  |  | 2 | 3 | 2 | 1 | 11 | 1 | 0 | 1 | 0 | 0 | 1 |  |  | 4 | 16 | 0.5 |
|  |  |  |  |  |  |  |  |  |  | 9.1% | 13.6% | 9.1% | 4.5% | 50.0% | 4.5% | 0.0% | 4.5% | 0.0% | 0.0% | 4.5% |  |  |  |  |  |
| ^3^Amoxicillin-clavulanic acid | 1 |  |  |  |  |  |  |  |  |  | 4 | 2 | 2 | 6 | 7 | 0 | 1 |  |  |  |  |  | 1 | 512 | 0.5 |
|  |  |  |  |  |  |  |  |  |  |  | 18.2% | 9.1% | 9.1% | 27.3% | 31.8% | 0.0% | 4.5% |  |  |  |  |  |  |  |  |
| Doxycycline | 0.5 |  |  |  |  |  |  |  |  | 2 | 5 | 4 | 1 | 0 | 3 | 1 | 3 | 1 | 0 | 0 | 0 |  | 1 | 64 | 0.5 |
|  |  |  |  |  |  |  |  |  |  | 9.1% | 22.7% | 18.2% | 4.5% | 0.0% | 13.6% | 4.5% | 13.6% | 4.5% | 0.0% | 0.0% | 0.0% |  |  |  |  |
| Enrofloxacin | 4 |  |  |  |  |  | 1 | 0 | 0 | 1 | 5 | 1 | 1 | 3 | 4 | 1 | 1 | 0 | 1 |  |  |  | 1 | 16 | 0.5 |
|  |  |  |  |  |  |  | 4.5% | 0.0% | 0.0% | 4.5% | 22.7% | 4.5% | 4.5% | 13.6% | 18.2% | 4.5% | 4.5% | 0.0% | 4.5% |  |  |  |  |  |  |
| Imipenem | 8 |  |  |  |  |  | 7 | 3 | 6 | 1 | 2 | 0 | 0 | 0 | 3 |  |  |  |  |  |  |  | 0.125 | 8 | 0.125 |
|  |  |  |  |  |  |  | 31.8% | 13.6% | 27.3% | 4.5% | 9.1% | 0.0% | 0.0% | 0.0% | 13.6% |  |  |  |  |  |  |  |  |  |  |
| ^4^Potentiated sulphonamide | 4 |  |  |  |  |  |  |  |  |  |  |  |  | 0 | 3 | 0 | 3 | 6 | 1 | 0 | 0 | 9 | 64 | 1024 | 0.25 |
|  |  |  |  |  |  |  |  |  |  |  |  |  |  | 0.0% | 13.6% | 0.0% | 13.6% | 27.3% | 4.5% | 0.0% | 0.0% | 40.9% |  |  |  |
| Tylosin | 64 |  |  |  |  |  |  |  |  |  | 2 | 9 | 1 | 3 | 0 | 0 | 1 | 1 | 0 | 3 | 1 | 1 | 1 | 256 | 2 |
|  |  |  |  |  |  |  |  |  |  |  | 9.1% | 40.9% | 4.5% | 13.6% | 0.0% | 0.0% | 4.5% | 4.5% | 0.0% | 13.6% | 4.5% | 4.5% |  |  |  |
| Tiamulin | 4 |  |  |  |  |  |  |  |  |  | 1 | 1 | 1 | 4 | 3 | 0 | 1 | 7 | 4 |  |  |  | 32 | 128 | 2 |
|  |  |  |  |  |  |  |  |  |  |  | 4.5% | 4.5% | 4.5% | 18.2% | 13.6% | 0.0% | 4.5% | 31.8% | 18.2% |  |  |  |  |  |  |
| Vancomycin | 32 |  |  |  |  |  |  |  |  | 2 | 7 | 7 | 6 |  |  |  |  |  |  |  |  |  | 1 | 2 | 2 |
|  |  |  |  |  |  |  |  |  |  | 9.1% | 31.8% | 31.8% | 27.3% |  |  |  |  |  |  |  |  |  |  |  |  |

^1^Clinical Laboratory Standard Institute (CLSI); ^2^Epidemiological cut-off value (EUCAST); ^3^2:1 ratio; ^4^trimetoprim-szulfametoxazol in ratio 1:19

**Supplementary Table 2** Frequency table of minimum inhibitory concentration (MIC) values for antimicrobial agents with established clinical breakpoints in *Enterococcus* isolates (*n*=74) from chickens in the Észak-Alföld region. The upper row for each compound displays the number of isolates, while the lower row shows the corresponding percentage. Vertical red lines indicate the clinical breakpoint, and green lines represent the epidemiological cutoff value (ECOFF).

| Antibiotics | ^1^Breakpoint | 0.001 | 0.002 | 0.004 | 0.008 | 0.016 | 0.031 | 0.063 | 0.125 | 0.25 | 0.5 | 1 | 2 | 4 | 8 | 16 | 32 | 64 | 128 | 256 | 512 | 1024 | MIC_50_ | MIC_90_ | ^2^ECOFF |
| --- | --- | --- | --- | --- | --- | --- | --- | --- | --- | --- | --- | --- | --- | --- | --- | --- | --- | --- | --- | --- | --- | --- | --- | --- | --- |
|  | µg/mL | | | | | | | | | | | | | | | | | | | | | | | | |
| Amoxicillin | 16 |  |  |  |  |  |  |  |  |  | 7 | 27 | 17 | 9 | 1 | 1 | 0 | 0 | 0 | 3 | 2 | 6 | 2 | 512 | - |
|  |  |  |  |  |  |  |  |  |  |  | 9.6% | 37.0% | 23.3% | 12.3% | 1.4% | 1.4% | 0.0% | 0.0% | 0.0% | 4.1% | 2.7% | 8.2% |  |  |  |
| ^3^Amoxicillin-clavulanic acid | 16 |  |  |  |  |  |  |  |  | 1 | 4 | 17 | 25 | 15 | 0 | 2 | 4 | 4 | 1 |  |  |  | 2 | 32 | - |
|  |  |  |  |  |  |  |  |  |  | 1.4% | 5.5% | 23.3% | 34.2% | 20.5% | 0.0% | 2.7% | 5.5% | 5.5% | 1.4% |  |  |  |  |  |  |
| Doxycycline | 16 |  |  |  |  |  |  |  | 3 | 5 | 1 | 1 | 1 | 17 | 22 | 17 | 3 | 3 |  |  |  |  | 8 | 16 | 1 |
|  |  |  |  |  |  |  |  |  | 4.1% | 6.8% | 1.4% | 1.4% | 1.4% | 23.3% | 30.1% | 23.3% | 4.1% | 4.1% |  |  |  |  |  |  |  |
| Enrofloxacin | 4 |  |  |  |  |  | 1 | 0 | 0 | 2 | 9 | 12 | 2 | 8 | 3 | 12 | 6 | 2 | 1 | 6 | 6 | 3 | 8 | 512 | - |
|  |  |  |  |  |  |  | 1.4% | 0.0% | 0.0% | 2.7% | 12.3% | 16.4% | 2.7% | 11.0% | 4.1% | 16.4% | 8.2% | 2.7% | 1.4% | 8.2% | 8.2% | 4.1% |  |  |  |
| Florfenicol | 8 |  |  |  |  |  |  |  |  |  |  |  |  | 24 | 35 | 7 | 2 | 1 | 1 | 2 | 1 |  | 8 | 16 | 8 |
|  |  |  |  |  |  |  |  |  |  |  |  |  |  | 32.9% | 47.9% | 9.6% | 2.7% | 1.4% | 1.4% | 2.7% | 1.4% |  |  |  |  |
| Imipenem | 16 |  |  |  |  |  |  |  | 2 | 3 | 8 | 16 | 21 | 16 | 3 | 1 | 0 | 0 | 0 | 2 | 1 |  | 2 | 4 | 4 |
|  |  |  |  |  |  |  |  |  | 2.7% | 4.1% | 11.0% | 21.9% | 28.8% | 21.9% | 4.1% | 1.4% | 0.0% | 0.0% | 0.0% | 2.7% | 1.4% |  |  |  |  |
| Neomycin | 1024 |  |  |  |  |  |  |  |  |  |  |  |  |  |  |  |  | 10 | 21 | 14 | 8 | 20 | 256 | 1024 | 256 |
|  |  |  |  |  |  |  |  |  |  |  |  |  |  |  |  |  |  | 13.7% | 28.8% | 19.2% | 11.0% | 27.4% |  |  |  |
| ^4^Potentiated sulphonamide | 64 |  |  |  |  |  |  |  |  |  |  | 8 | 2 | 13 | 9 | 3 | 2 | 1 | 0 | 1 | 3 | 31 | 32 | 1024 | - |
|  |  |  |  |  |  |  |  |  |  |  |  | 11.0% | 2.7% | 17.8% | 12.3% | 4.1% | 2.7% | 1.4% | 0.0% | 1.4% | 4.1% | 42.5% |  |  |  |
| Tylosin | 8 |  |  |  |  |  |  |  |  |  |  |  | 3 | 2 | 1 | 1 | 0 | 1 | 2 | 7 | 28 | 28 | 512 | 1024 | - |
|  |  |  |  |  |  |  |  |  |  |  |  |  | 4.1% | 2.7% | 1.4% | 1.4% | 0.0% | 1.4% | 2.7% | 9.6% | 38.4% | 38.4% |  |  |  |
| Vancomycin | 32 |  |  |  |  |  |  |  |  | 1 | 0 | 12 | 12 | 10 | 14 | 2 | 1 | 0 | 2 | 10 | 4 | 5 | 8 | 512 | 4 |
|  |  |  |  |  |  |  |  |  |  | 1.4% | 0.0% | 16.4% | 16.4% | 13.7% | 19.2% | 2.7% | 1.4% | 0.0% | 2.7% | 13.7% | 5.5% | 6.8% |  |  |  |

^1^Clinical Laboratory Standard Institute (CLSI); ^2^Epidemiological cut-off value (EUCAST); ^3^2:1 ratio; ^4^trimetoprim-szulfametoxazol in ratio 1:19

**Supplementary Table 3** Frequency table of minimum inhibitory concentration (MIC) values for antimicrobial agents with established clinical breakpoints in *Escherichia coli* isolates (*n*=71) from chickens in the Észak-Alföld region. The upper row for each compound displays the number of isolates, while the lower row shows the corresponding percentage. Vertical red lines indicate the clinical breakpoint, and green lines represent the epidemiological cutoff value (ECOFF).

| Antibiotics | ^1^Breakpoint | 0.001 | 0.002 | 0.004 | 0.008 | 0.016 | 0.031 | 0.063 | 0.125 | 0.25 | 0.5 | 1 | 2 | 4 | 8 | 16 | 32 | 64 | 128 | 256 | 512 | 1024 | MIC_50_ | MIC_90_ | ^2^ECOFF |
| --- | --- | --- | --- | --- | --- | --- | --- | --- | --- | --- | --- | --- | --- | --- | --- | --- | --- | --- | --- | --- | --- | --- | --- | --- | --- |
|  | µg/mL | | | | | | | | | | | | | | | | | | | | | | | | |
| Amoxicillin | 32 |  |  |  |  |  |  |  |  |  |  |  |  | 2 | 0 | 1 | 2 | 3 | 1 | 2 | 7 | 53 | 1024 | 1024 | 8 |
|  |  |  |  |  |  |  |  |  |  |  |  |  |  | 2.8% | 0.0% | 1.4% | 2.8% | 4.2% | 1.4% | 2.8% | 9.9% | 74.6% |  |  |  |
| ^3^Amoxicillin-clavulanic acid | 32 |  |  |  |  |  |  |  |  |  |  |  |  |  | 7 | 4 | 16 | 16 | 21 | 7 |  |  | 64 | 128 | 8 |
|  |  |  |  |  |  |  |  |  |  |  |  |  |  |  | 9.9% | 5.6% | 22.5% | 22.5% | 29.6% | 9.9% |  |  |  |  |  |
| Ceftriaxone | 4 |  |  |  |  |  | 16 | 16 | 5 | 17 | 8 | 2 | 0 | 2 | 0 | 1 | 0 | 0 | 0 | 1 | 0 | 3 | 0.125 | 1 | 0.125 |
|  |  |  |  |  |  |  | 22.5% | 22.5% | 7.0% | 23.9% | 11.3% | 2.8% | 0.0% | 2.8% | 0.0% | 1.4% | 0.0% | 0.0% | 0.0% | 1.4% | 0.0% | 4.2% |  |  |  |
| Doxycycline | 16 |  |  |  |  |  |  |  |  |  | 5 | 9 | 8 | 10 | 2 | 8 | 19 | 7 | 2 | 1 |  |  | 16 | 64 | 8 |
|  |  |  |  |  |  |  |  |  |  |  | 7.0% | 12.7% | 11.3% | 14.1% | 2.8% | 11.3% | 26.8% | 9.9% | 2.8% | 1.4% |  |  |  |  |  |
| Enrofloxacin | 2 |  |  |  |  |  |  |  |  | 1 | 10 | 9 | 6 | 3 | 6 | 19 | 10 | 1 | 1 | 1 | 2 | 2 | 16 | 128 | 0.125 |
|  |  |  |  |  |  |  |  |  |  | 1.4% | 14.1% | 12.7% | 8.5% | 4.2% | 8.5% | 26.8% | 14.1% | 1.4% | 1.4% | 1.4% | 2.8% | 2.8% |  |  |  |
| Florfenicol | 16 |  |  |  |  |  |  |  |  |  |  |  |  | 2 | 5 | 16 | 6 | 21 | 1 | 8 | 12 |  | 64 | 512 | 16 |
|  |  |  |  |  |  |  |  |  |  |  |  |  |  | 2.8% | 7.0% | 22.5% | 8.5% | 29.6% | 1.4% | 11.3% | 16.9% |  |  |  |  |
| Imipenem | 4 |  |  |  |  |  |  | 7 | 28 | 14 | 14 | 1 | 0 | 1 | 3 | 3 |  |  |  |  |  |  | 0.25 | 8 | 0.5 |
|  |  |  |  |  |  |  |  | 9.9% | 39.4% | 23.9% | 19.7% | 1.4% | 0.0% | 1.4% | 4.2% | 4.2% |  |  |  |  |  |  |  |  |  |
| Colistin | 2 |  |  |  |  |  | 2 | 10 | 3 | 11 | 6 | 1 | 0 | 0 | 1 | 1 | 1 | 6 | 5 | 2 | 6 | 16 | 32 | 1024 | 2 |
|  |  |  |  |  |  |  | 2.8% | 14.1% | 4.2% | 15.5% | 8.5% | 1.4% | 0.0% | 0.0% | 1.4% | 1.4% | 1.4% | 8.5% | 7.0% | 2.8% | 8.5% | 22.5% |  |  |  |
| Neomycin | 32 |  |  |  |  |  |  |  |  |  |  |  | 1 | 10 | 0 | 7 | 22 | 15 | 9 | 1 | 3 | 3 | 32 | 512 | 8 |
|  |  |  |  |  |  |  |  |  |  |  |  |  | 0.0% | 14.1% | 0.0% | 9.9% | 31.0% | 21.1% | 12.7% | 1.4% | 4.2% | 4.2% |  |  |  |
| ^4^Potentiated sulphonamide | 4 |  |  |  |  |  |  |  |  |  |  |  | 16 | 17 | 10 | 8 | 2 | 0 | 0 | 1 | 0 | 17 | 8 | 1024 | 0.5 |
|  |  |  |  |  |  |  |  |  |  |  |  |  | 22.5% | 23.9% | 14.1% | 11.3% | 2.8% | 0.0% | 0.0% | 1.4% | 0.0% | 23.9% |  |  |  |
| Spectinomycin | 128 |  |  |  |  |  |  |  |  |  |  |  |  |  |  |  | 11 | 12 | 22 | 7 | 7 | 12 | 128 | 1024 | 64 |
|  |  |  |  |  |  |  |  |  |  |  |  |  |  |  |  |  | 15.5% | 16.9% | 31.0% | 9.9% | 9.9% | 16.9% |  |  |  |

^1^ Clinical Laboratory Standard Institute (CLSI); ^2^Epidemiological cut-off value (EUCAST); ^3^2:1 ratio; ^4^trimetoprim-szulfametoxazol in ratio 1:19
